# Generation of an RCVRN-eGFP Reporter hiPSC Line by CRISPR/Cas9 to Monitor Photoreceptor Cell Development and Facilitate the Cell Enrichment for Transplantation

**DOI:** 10.3389/fcell.2022.870441

**Published:** 2022-04-28

**Authors:** Yuanyuan Guan, Yuan Wang, Dandan Zheng, Bingbing Xie, Ping Xu, Guanjie Gao, Xiufeng Zhong

**Affiliations:** State Key Laboratory of Ophthalmology, Zhongshan Ophthalmic Center, Sun Yat-sen University, Guangzhou, China

**Keywords:** human induced pluripotent stem cells, reporter line, retinal organoids, recoverin, photoreceptor development, transcriptome, enrichment

## Abstract

Stem cell-based cell therapies are considered to be promising treatments for retinal disorders with dysfunction or death of photoreceptors. However, the enrichment of human photoreceptors suitable for transplantation has been highly challenging so far. This study aimed to generate a photoreceptor-specific reporter human induced pluripotent stem cell (hiPSC) line using CRISPR/Cas9 genome editing, which harbored an enhanced green fluorescent protein (*eGFP*) sequence at the endogenous locus of the pan photoreceptor marker recoverin (*RCVRN*)*.* After confirmation of successful targeting and gene stability, three-dimensional retinal organoids were induced from this reporter line. The RCVRN-eGFP reporter faithfully replicated endogenous protein expression of recoverin and revealed the developmental characteristics of photoreceptors during retinal differentiation. The RCVRN-eGFP specifically and steadily labeled photoreceptor cells from photoreceptor precursors to mature rods and cones. Additionally, abundant eGFP-positive photoreceptors were enriched by fluorescence-activated cell sorting, and their transcriptome signatures were revealed by RNA sequencing and data analysis. Moreover, potential clusters of differentiation (CD) biomarkers were extracted for the enrichment of photoreceptors for clinical applications, such as CD133 for the positive selection of photoreceptors. Altogether, the RCVRN-eGFP reporter hiPSC line was successfully established and the first global expression database of recoverin-positive photoreceptors was constructed. These achievements will provide a powerful tool for dynamically monitoring photoreceptor cell development and purification of human photoreceptors, thus facilitating photoreceptor cell therapy for advanced retinal disorders.

## Introduction

Vision is the most considerable sense for humans with almost 30% of the sensory input to the brain coming from the retina (Sharma et al., 2020). The retina provides an exquisitely sensitive vision that relies on light-sensing photoreceptors, which constitute about 80% of cells in the human retina with two subtypes: rods and cones (Burger et al., 2021). Photoreceptors capture visual information first, sense photons of light, and initiate phototransduction (Molday and Moritz, 2015). Consequently, any dysfunction or death of photoreceptors can lead to devastating sight loss in retinal disorders (RDs), including age-related macular degeneration (AMD) and retinitis pigmentosa (RP). AMD is thought to globally affect over 8 million people (Flaxman et al., 2017) and RP is the most common retinal inherited disease, approximately 1 in 5,000 worldwide (Huang, 2018). Currently, clinical treatments are limited to delaying the progress of RDs, such as pharmacotherapy and gene therapy (Scholl et al., 2016; Miller et al., 2017). But there is no available way to restore dysfunctional photoreceptors once substantial photoreceptor damage occurs. Cell therapies are considered to be promising treatments for advanced RDs (West et al., 2020).

In the past decades, cell transplantation into the subretinal space of the eye has been attempted to replace the lost photoreceptors for vision recovery of the late-stage RDs in animal models. Various types of donor cells, such as neural progenitors (Jones et al., 2016; Bian et al., 2020), retinal progenitor cells (RPCs) (Zou et al., 2019), and photoreceptors (Santos-Ferreira et al., 2017; Llonch et al., 2018), have been used in the transplantation and showed that photoreceptors might be the optimal cell sources for the cell replacement of late-stage RDs. However, there are still many hurdles in photoreceptor transplantation, such as the short-term survival of donor cells, poor cell integration, and functional vision recovery (Gonzalez-Cordero et al., 2013; Decembrini et al., 2014), which are subject to many factors. Previous studies point out that the ontogenic stage of photoreceptors is a vital factor affecting the success of transplantation (Llonch et al., 2018). Compared to the donor cells from mature rods and cones, the graft of the postmitotic photoreceptor precursors into subretinal space of animal models achieved better outcomes including improved cell survival, integration, and even visual function (MacLaren et al., 2006; Lakowski et al., 2010; West et al., 2010; Pearson et al., 2012; Singh et al., 2013; Santos-Ferreira et al., 2015). However, recent works indicated these outcomes might be achieved by the cytoplasmic material transferring instead of the true donor cell integration (Pearson et al., 2016; Santos-Ferreira et al., 2016; Singh et al., 2016). Therefore, it is still unclear which developmental stages of donor photoreceptors are suitable for grafts with the best outcomes. To clarify this issue, the prerequisite is to acquire a large number of donor photoreceptors, photoreceptors at distinct ontogenetic stages in particular.

In previous preclinical research, donor photoreceptors were isolated mainly from embryonic or neonatal mice retinas (Santos-Ferreira et al., 2017). However, the corresponding donor cells from human retinas pose not only significant ethical concerns but also severe limitations on the availability of material. A breakthrough for acquiring human photoreceptors has been made with three-dimensional (3D) retinal organoids (ROs) derived from human pluripotent stem cells (hPSCs), including human induced pluripotent stem cells (hiPSCs) and human embryonic stem cells (hESCs) (Nakano et al., 2012; Zhong et al., 2014; Li et al., 2018; Capowski et al., 2019; Kruczek and Swaroop, 2020). However, how to purify photoreceptors from hPSCs-derived ROs is highly challenging. Although single or combined cell surface markers, such as clusters of differentiation (CD) biomarkers, CD26+, CD73+, CD133+, and CD15− (Lakowski et al., 2015; Welby et al., 2017; Gagliardi et al., 2018; Lakowski et al., 2018), have been tried to enrich photoreceptors from mouse or human retina by fluorescence-activated cell sorting (FACS) or magnetic-activated cell sorting (MACS), markers with high specificity are still unavailable. In addition, some photoreceptor marker reporters were also constructed to label photoreceptors, such as CRX reporter for developing photoreceptors (Kaewkhaw et al., 2015; Collin et al., 2016; Phillips et al., 2018b), NRL reporter for rods (Phillips et al., 2018a) and L/M opsin reporter for mature red/green cones (Welby et al., 2017), which could help purify subtypes of photoreceptors at specific developmental stages. However, in most of the advanced RDs, both rods and cones are always lost. Therefore, it is desirable to have a reporter that can simply cover the entire developmental stages of photoreceptors for further evaluation of the optimal donor cells in preclinical cell therapy studies.

Recoverin, a calcium-binding protein encoded by the gene *RCVRN*, is a well-known pan photoreceptor marker in the mammalian retina (Ames and Ikura, 2002). In the human fetal retina, recoverin starts to present in photoreceptor precursors by 13 fetal weeks and is widely expressed in both cones and rods afterward (Yan and Wiechmann, 1997). In the adult retina, recoverin locates primarily in the photoreceptor outer segment and plays an important role in phototransduction (Chen et al., 1995; Chen et al., 2012; Morshedian et al., 2018). Hence, the expression of recoverin covers almost all the developmental stages and subtypes of photoreceptors, which makes it a suitable candidate reporter for labeling and purifying all photoreceptors.

Here, we successfully established an RCVRN-reporter hiPSC line harboring an enhanced green fluorescent protein (*eGFP*) sequence at the endogenous locus of the *RCVRN* using CRISPR/Cas9 gene-editing technique and dynamically monitored the photoreceptor development in live ROs. Additionally, recoverin positive photoreceptors were purified and the transcriptome profiles of photoreceptors were revealed. Moreover, the potential CD biomarkers for photoreceptor selection were extracted. The RCVRN-eGFP reporter hiPSCs can be a powerful tool to investigate the development, cellular and molecular characteristics of photoreceptors, thus facilitating the enrichment of photoreceptors for future clinical therapies of RDs. The datasets we established will also serve as a reference for studying genetic regulation underlying human photoreceptor development and diseases.

## Materials and Methods

### Human iPSC Culture

The BC1 hiPSC line was a gift from Professor Linzhao Cheng (University of Science and Technology of China) (Chou et al., 2011). HiPSCs were cultured in mTeSR1 medium (Stemcell Technologies) on Matrigel (Corning) coated 6-well plates, and passaged every 5–7 days at about 80% confluence according to our previously published protocol (Guan et al., 2021).

### Generation of RCVRN-eGFP Reporter hiPSC Line

The single guide RNA (sgRNA) was designed by the online website (http://crispor.tefor.net/). The sgRNA (ACA​GCT​GAA​CAG​TTG​GCA​TC), which targeted near the TGA stop codon of exon 3 of *RCVRN* and was predicted to have over 50% cleavage activity, was cloned into pSpCas9 (BB)-2A-Puro (PX459; Addgene) to generate the CRISPR/Cas9 guide-carrying plasmids PX459-RCVRN-sgRNA. For targeting plasmid construction, first, *P2A-eGFP* fragment was amplified from pBluescript-LA-P2A-eGFP-RA (*VSX2*) (a gift from Professor Mengqing Xiang, Zhongshan Ophthalmic Center, Sun Yat-sen University, China), and the left and right homologous arms were amplified from the BC1 hiPSC genome. Second, these fragments were further amplified by primers with infusion homologous sequence. Third, these three fragments were linked together with pMD19-T vector (Takara; 6013) by in-Fusion^®^ HD Cloning Kit (Takara; 639648). The specific structure of the donor plasmid was shown in Supplementary Figure S1, and the PCR primers for targeting plasmid construction were listed in Supplementary Table S1.

Then, the BC1 hiPSCs were digested into single cells using an accutase digestive enzyme (Gibco). Approximately 1 × 10^6^ cells in 100 μl Buffer R solution were mixed with 10 μg guide-carrying plasmids and 10 μg targeting plasmids according to the manufacturer’s instruction (Neon; MPK10025). Then the mixture was electroporated with Neon™ transfection system (MPK5000) under the manufacturer’s recommended procedure: 1,100 v; 10 ms; 3 pulses. After electroporation, cells were transferred into a Matrigel-coated 24-well plate containing mTesR1 medium supplemented with 10 mM Y-27632 (Stemcell Technologies) in an incubator at 37°C and 5% CO_2_. Three days later, the cells were treated with 0.4 μg/ml puromycin for 7 days to select the resistant clones. Finally, the selected clones were further confirmed by polymerase chain reaction (PCR) and by Sanger sequencing.

### PCR and Sanger Sequencing

The genomic DNA was extracted using an Animal Genomic DNA Quick Extraction Kit (Beyotime, China) according to the manufacturer’s protocol. PCR was performed using 2× Taq MasterMix (Takara). The following PCR primers were used: *RCVRN,* Forward, AGA​TGT​CAG​CCT​CAT​CGC​AG; Reverse, CAT​CGC​AAC​ACA​GCC​TTG​TC. The forward primer was designed to target the 5′ upstream outside of the left homologous arm so that the correct insertion could be identified by one round of PCR experiment. The PCR conditions were: 95°C for 3 min, followed by 35 cycles of 95°C for 15 s, 55°C for 15 s and 72°C for 1 min, and the final cycle of 72°C for 5 min. The PCR products were resolved on a 1.5% agarose gel by electrophoresis. Sanger sequencing was performed by Sangon Biotech (Shanghai, China) Co.

### Short Tandem Repeats Authentication

About 2 × 10^6^ reporter hiPSCs were collected and sent to Nanjing Cobioer Biosciences Company (Nanjing, China) for short tandem repeats (STR) analysis. The STR result was compared with that of the parental hiPSCs.

### Generation of 3D ROs from the Reporter hiPSCs

The differentiation of RCVRN-eGFP hiPSCs into ROs was performed according to our previously published protocol (Guan et al., 2021). Briefly, hiPSCs were dissociated and cultured in suspension at day (D) 0 to form embryoid bodies (EBs). On D1 to D3, EBs were gradually switched to neural induction medium (NIM) containing DMEM/F12 (1:1) (Gibco), 1% N2 supplement (Gibco), 1% minimum essential media nonessential amino acids (MEM NEAA, Gibco), and 2 mg/ml heparin (Sigma-Aldrich). During D5 to D7, EBs were seeded on Matrigel-coated dishes with NIM. The medium was changed to retinal differentiation medium (RDM) containing DMEM/F12 (3:1), 2% B27 (without vitamin A, Gibco), 1% MEM NEAA, and 1% Antibiotic-Antimycotic (Gibco) since D16. Typical optical vesicles (OVs) appeared around D25 with adjacent pigmented retinal pigment epithelium cells (RPEs). They were mechanically picked up and switched to suspension culture for the development and maturation of ROs. For long-term culture, the RDM was supplemented with 10% fetal bovine serum (FBS, Natocor), 100 μM Taurine (Sigma-Aldrich), and 2 mM GlutaMAX (Gibco) since D42, and then B27 was replaced with 1% N2 since D90. The medium was changed twice a week. Bright-field and fluorescence images were taken using an Axio observer 7 microscope (Zeiss).

### Immunofluorescence Analysis

The hiPSCs were fixed using 4% paraformaldehyde (PFA) for 10 min at room temperature (RT). ROs were fixed using 4% PFA for 30 min at RT and dehydrated in gradient sucrose solutions at 4°C, 6.25% for 30 min, 12.5% for 30 min, and 25% overnight before embedding in O.C.T compound. Cryosections were mounted on slides for immunofluorescence (IF) staining according to previously described protocols (Li et al., 2019). Briefly, cells or sections were blocked and permeabilized with 0.25% Triton X-100 and 10% donkey serum for 1 h at RT, then incubated with primary antibodies at 4°C overnight and incubated with the corresponding secondary antibodies for 1 h at RT in the dark. DAPI (Dojindo) was used to counterstain nuclei. All antibodies used are listed in Supplementary Table S2. IF images were taken with an LSM 880 confocal microscope (Zeiss) and an Axio Scan Z1 (Zeiss). Images were processed in Zen 2.3 (Zeiss) and Adobe Illustrator CS6 (Adobe Systems Incorporated).

### Karyotype Analysis

Karyotype analysis was performed using G-band staining of chromosomes. The reporter hiPSCs were cultured on Matrigel-coated 6-well plates with mTeSR1 medium until reaching 50% confluences. Colchicine was added into the culture medium to a final concentration of 0.8 μg/ml. The reporter hiPSCs were cultured for another 2.5 h. Then the cells were digested with 0.25% Trypsin (Invitrogen) for 1 min at 37°C, collected, and sent to Guangzhou Daan Clinical Laboratory Center (Guangzhou, China) for further analysis. Chromosomes were classified according to the International System for Human Cytogenetic Nomenclature (Simons et al., 2013).

### Teratoma Formation Assay

1.5–2.5 × 10^6^ reporter hiPSCs with 30% Matrigel were injected intramuscularly into the hind limb of 6-week old immunocompromised NOD-SCID mice. Animals were monitored each week and after 5–7 weeks, teratomas were dissected and fixed in formalin, embedded in paraffin, sectioned, and stained with Hematoxylin and Eosin. Images were taken with an Axio Scan Z1 (Zeiss). The study design and experimental protocols were approved by the Animal Ethics Committee of the Zhongshan Ophthalmic Center, Sun Yat-sen University. All experimental procedures involving animals adhered to the Association Research in Vision and Ophthalmology Statement for the Use of Animals in Ophthalmic and Vision Research.

### Quantitative Reverse Transcription-PCR

Total RNA was extracted using TRIzol Reagent (Invitrogen), and potential DNA contamination was removed by PrimeScript RT reagent Kit with gDNA Eraser (Perfect Real Time) (Takara). The quality of RNA was evaluated using a SMA4000 Ultra Micro Spectrophotometer (Merinton). Reverse transcription was performed using the PrimeScript RT reagent Kit with gDNA Eraser (Perfect Real Time) (Takara). Quantitative PCR was performed with Hieff UNICON qPCR SYBR Green Master Mix (YeaSen) using an ABI7300 fluorescence quantitative PCR instrument (Thermo Fisher Scientific) following the manufacturer’s instructions. 2^−ΔΔCt^ method was used to analyze data. The BC1 hiPSCs were used as references and GAPDH was used as the internal control. All primer sequences are listed in Supplementary Table S3.

### FACS and Data Analysis

ROs derived from RCVRN-eGFP reporter line were collected at D150 and mechanically separated into small pieces by a needle with 1 ml syringe. Then the clumps were dissociated into single cells by a papain dissociation system (Worthington Biochemical) according to the manufacturer’s instructions. Cell suspensions were resuspended in sorting buffer (PBS containing 1 mM EDTA (Gibco), 2% [vol/vol] FBS). Cells were sorted at 4°C by BD FACSAria III Cytometer (BD Biosciences). Both eGFP-positive (eGFP+) and negative (eGFP-) cells were separately collected in collecting buffer [50% culture medium and 50% (vol/vol) FBS]. BD FACSDiva v8.0.1 software (BD Biosciences) was used for data file collection and data analysis.

### RNA-Seq and Data Analysis

Cells from D150-ROs derived from the RCVRN-eGFP reporter hiPSCs were used to perform RNA-seq. Unsorted, FACS-sorted eGFP+ and eGFP- cells (30 ROs per experiment, 2 independent experiments) were respectively collected in TRIzol reagent (Invitrogen) and stored in a −80°C freezer until submitted to Gene Denovo Biotechnology Co. (Guangzhou, China) for RNA extraction, library preparation, sequencing and data analyses. Total RNA was extracted using TRIzol reagent kit (Invitrogen) according to the manufacturer’s protocol. The quality of the RNA was assessed by an Agilent 2100 Bioanalyzer (Agilent Technologies) and checked using RNase free agarose gel electrophoresis. Oligo (dT) beads were used to isolate the poly mRNA from the total RNA. Then the enriched mRNA was subjected to RNA-seq library construction.

The mapped reads of each sample were assembled by StringTie v1.3.1 (Pertea et al., 2015). Gene expression levels were quantified by software RSEM (Li and Dewey, 2011). For each transcription, a fragment per kilobase of transcript per million mapped reads (FPKM) value was calculated to quantify its expression, abundance, and variations. Correlation analysis was performed by R. correlation of two parallel experiments. Differential expression analysis was performed by DESeq2 (1.18.0) between two different groups and by edgeR between two samples (Robinson et al., 2010). Unless otherwise stated, the genes/transcripts with the parameter of false discovery rate (FDR) below 0.05 and absolute fold change (FC) > 2 were considered differentially expressed genes (DEGs). The Gene Ontology (GO) enrichment analysis with a Q-value < 0.05 was defined as significantly enriched.

### Statistical Analysis

All data are presented as mean ± SD. Comparisons between two groups were analyzed using a two-tailed Student’s t-test. *p*-value < 0.05 was considered statistically significant. When more than three groups, a two-way ANOVA analysis was performed. A *p*-value < 0.05 was considered significant. GraphPad Prism version 9.2.0 (GraphPad Software) was used for the calculation of probability values. For statistical analysis of RNA-seq, see RNA-seq and data analysis for more details.

## Results

### Generation and Characterization of RCVRN-eGFP hiPSC Line

**FIGURE 1:**
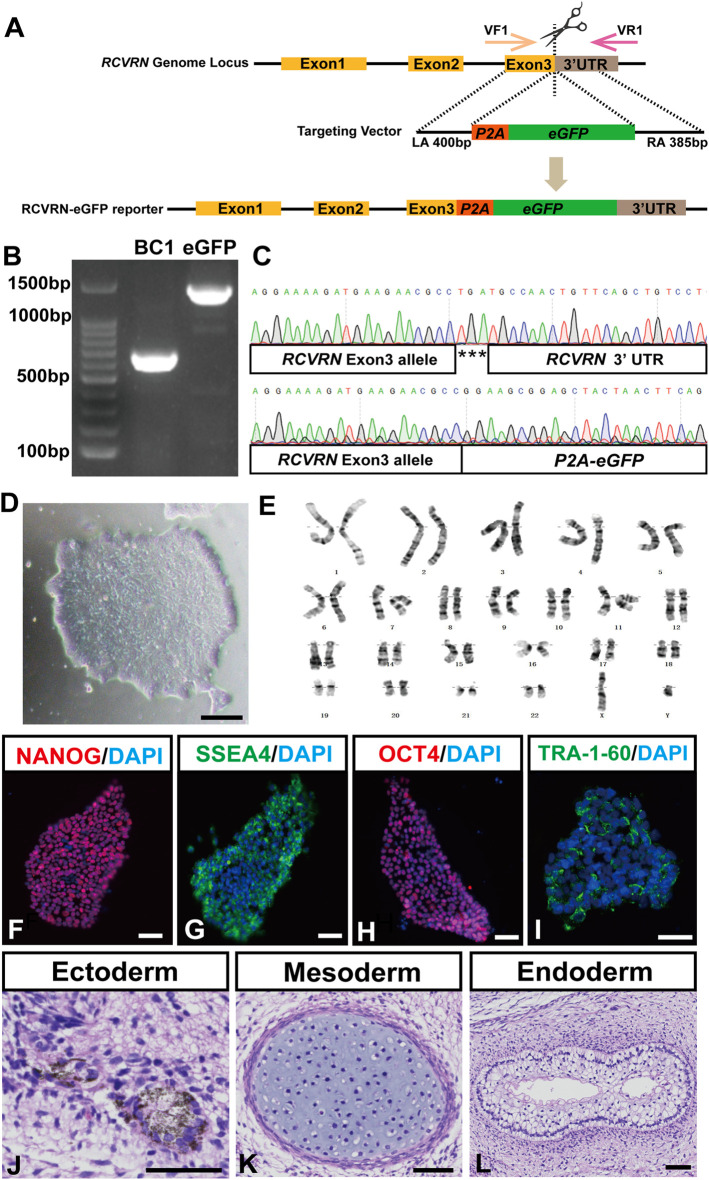
Generation and characterization of the RCVRN-eGFP reporter hiPSCs. **(A)** Scheme of CRISPR/Cas9 mediated genome editing used to generate the RCVRN-eGFP reporter. VF1, validation forward primer 1; VR1, validation reverse primer 1; UTR, untranslated region; LA, left arm; *P2A*, porcine teschovirus 2A self-cleaving peptide; *eGFP*, enhanced green fluorescence protein; RA, right arm. **(B)** Verification of the eGFP cassette insertion by PCR amplification of genomic DNA. BC1, parental hiPSCs; eGFP, hiPSCs with eGFP cassette insertion. **(C)** Sanger sequencing of the reporter hiPSCs showed the correct insertion of the *P2A-eGFP* at the endogenous *RCVRN* locus. **(D)** A typical clone of the reporter hiPSCs. **(E)** G-banding analysis of the reporter hiPSCs. **(F–I)** Immunofluorescence images showed the expression of the pluripotency markers NANOG, SSEA4, OCT4, and TRA-1-60 in the reporter hiPSCs. Nuclei were stained with DAPI. **(J–L)** Histological staining showed three germ layers in teratomas derived from the reporter hiPSCs: ectoderm (pigment cells), mesoderm (cartilage tissue), and endoderm (glandular tissue). Scale bars, 50 μm (D, F-L).

To generate the RCVRN-reporter hiPSC line, a viral *P2A-eGFP* fused sequence was designed to insert before the *RCVRN* exon 3 TGA stop codon of the genome in the BC1 hiPSC line (Figure 1A). After selection of puromycin resistance, one of the surviving clones was verified as the correctly targeted clone by PCR and Sanger sequencing (Figures 1B,C). This clone was expanded and confirmed with typical hiPSC morphology and normal karyotype (Figures 1D,E). STR analysis displayed the genetic matches between the reporter and the parental hiPSCs.

Subsequently, the pluripotency and multi-lineage differentiation ability of the reporter hiPSCs were characterized. The reporter hiPSCs expressed typical pluripotency marker proteins NANOG, SSEA4, OCT4, and TRA-1-60 (Figures 1F–I). qRT-PCR further showed that the reporter hiPSCs had similar mRNA expression levels in pluripotency markers to the parental ones, including OCT4, SOX2, NANOG, GDF3, and DNMT3B (Supplementary Figure S2A). Teratoma formation assays confirmed the multi-lineage differentiation ability of the reporter hiPSCs that differentiated into three germ layers, including pigment cells (ectoderm), cartilage (mesoderm), and gut-like epithelium (endoderm) (Figures 1J–L). Finally, the reporter hiPSCs were guided to induce ROs with our reported protocol (Guan et al., 2021). D28 after differentiation, 3D ROs with thick and transparent neural retina-like structures (NRs) and pigmented RPEs spontaneously formed, demonstrating the retinal differentiation ability of the reporter hiPSCs (Supplementary Figures S2B–E). Altogether, an RCVRN-eGFP reporter hiPSC line was successfully established and had the ability to differentiate into human ROs for further investigations.

### Validating the Faithfulness and Robustness of RCVRN-eGFP Reporter in ROs

**FIGURE 2:**
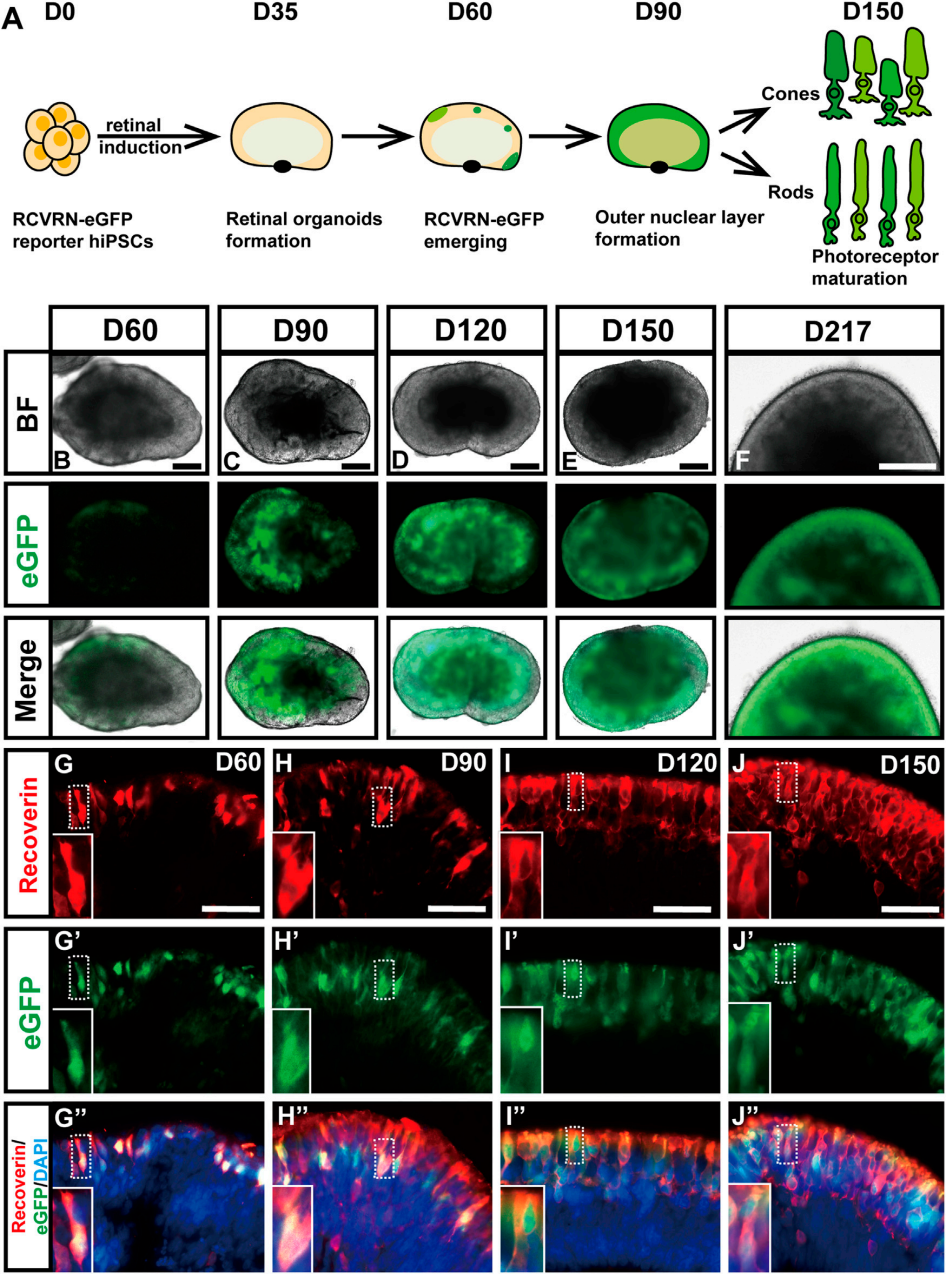
Validating the faithfulness and robustness of RCVRN-eGFP during retinal induction. **(A)** Timeframe of the expression of the RCVRN-eGFP (eGFP) in retinal organoids (ROs). **(B–F)** Bright-field (BF) and fluorescent images showed the expression patterns of eGFP in ROs over time. **(G–J”)** Immunofluorescence images showed the co-localization of eGFP and endogenous recoverin in ROs at different time points. Nuclei were stained with DAPI. Scale bars, 200 μm (B–F); 50 μm (G-J″).

To evaluate whether the RCVRN-eGFP reporter functions well, the protein expression of recoverin in the reporter hiPSC-derived ROs was dynamically monitored under the fluorescence microscope (Figure 2). RCVRN-eGFP expression initially appeared in patches of cells in the NR layers during D50 to D60 (Figure 2B), gradually increasing and becoming abundant over time (Figures 2C–F). The percentage of RCVRN-eGFP-positive (eGFP+) ROs was 63.75 ± 8.54% at D60 (5 independent experiments; 20 ROs per experiment) and increased to 100 ± 0% by D73. The eGFP+ ROs developed and matured over time. Outer segment-like protrusions appeared on the outmost side of the NR layers and remained apparent up to D217, the latest time point observed (Figure 2F). In addition, no RCVRN-eGFP expression was identified in the RPE of the ROs (Supplementary Figures S3A–C). IF staining with anti-recoverin antibody demonstrated that the eGFP expression was co-localized with recoverin in ROs at all time points tested (Figure 2G–J″). These data suggested that the RCVRN-eGFP reporter could robustly and accurately monitor the expression of recoverin in the reporter ROs.

### RCVRN-eGFP Sequentially Labels Photoreceptor Precursors and Mature Rods and Cones in Developing ROs

**FIGURE 3:**
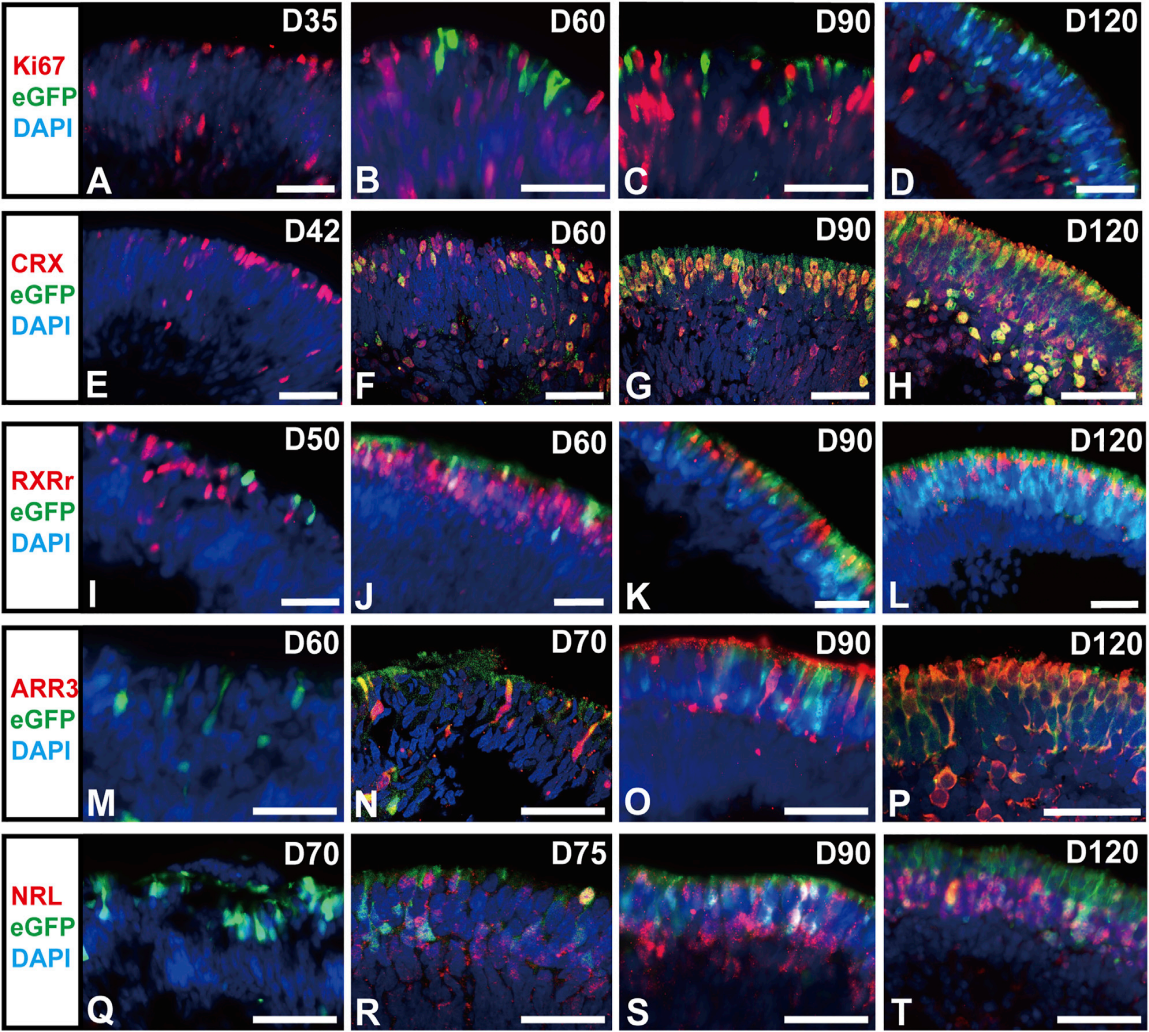
RCVRN-eGFP labels postmitotic photoreceptor precursors in early-stage retinal organoids. **(A–D)** Immunofluorescence (IF) images showed RCVRN-eGFP+ cells did not express the proliferation marker Ki67 in the reporter retinal organoids (ROs). **(E–T)** IF images showed the expression of photoreceptor precursor marker CRX, cone precursor marker RXRr and ARR3, and rod precursor marker NRL in the eGFP+ cells in early-stage ROs younger than D120. Nuclei were stained with DAPI. Scale bars, 50 μm **(A–T)**.

Next, the identity of the RCVRN-eGFP+ cells was explored in ROs at different developmental stages. They started to appear in NR of ROs since D50 after differentiation and self-formed an outer nuclear layer (ONL)-like structure in NRs over time (Figure 3). All eGFP+ cells were negative for proliferation marker Ki67 during the whole differentiation progress of photoreceptors, declaring that they were in postmitosis (Figures 3A–D, Supplementary Figure S4). In early-stage ROs aged younger than D120, the eGFP+ cells expressed CRX, a cone-rod photoreceptor precursor marker (Assawachananont et al., 2018; Yamamoto et al., 2020) (Figures 3E–H, Supplementary Figure S5). Moreover, eGFP+ cells progressively expressed both cone precursor markers RXRr (Figures 3I–L, Supplementary Figure S6) and Arrestin3 (ARR3) (Figures 3M–P, Supplementary Figure S7), and rod precursor marker NRL (Figures 3Q–T, Supplementary Figure S8). All these data elucidated that RCVRN-eGFP represented postmitotic photoreceptor precursors including both cone and rod precursors in early-stage ROs.

**FIGURE 4:**
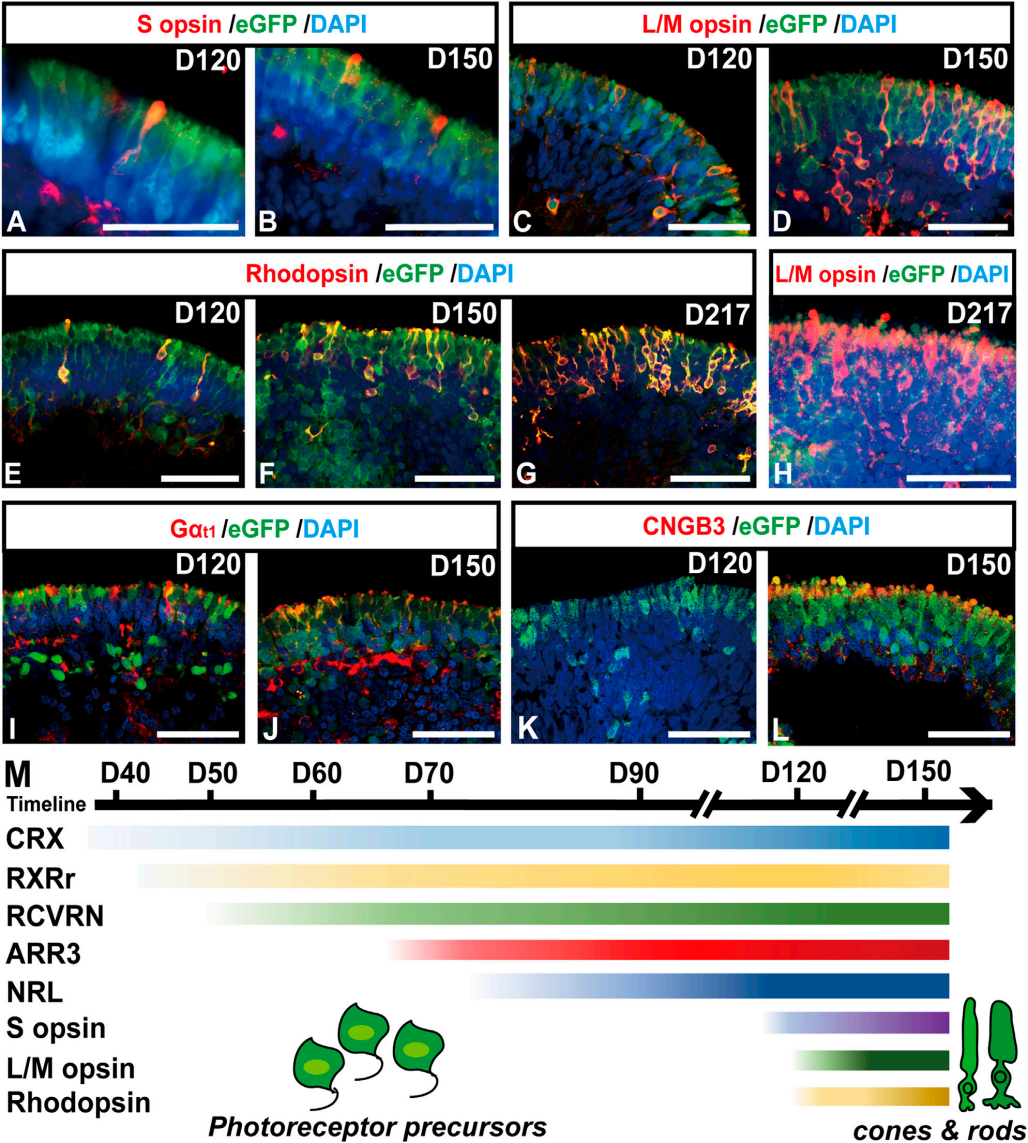
RCVRN-eGFP labels both mature cones and rods in late-stage retinal organoids. **(A–L)** Immunofluorescence images showed the expression of blue cone marker S opsin, red/green cone marker L/M opsin, rod marker Rhodopsin, α-subunit of rod transducin (Gα_t1_), and cone-specific phototransduction protein CNGB3 in eGFP+ cells in late-stage retinal organoids older than D120. **(M)** Scheme outlined the temporal expression patterns of photoreceptor-specific markers during retinal differentiation in retinal organoids. Nuclei were stained with DAPI. Scale bars, 50 μm (A–L).

In late-stage ROs older than D120, relatively mature photoreceptor subtypes, one rod and two cones (Xiao and Hendrickson, 2000; Hendrickson et al., 2008), started to present in ROs (Figure 4). A subpopulation of eGFP+ cells highly expressed blue cone marker S opsin (Figures 4A,B, Supplementary Figures S9A,B″), red/green cone marker L/M opsin (Figures 4C,D, Supplementary Figures S9C,D″), and rod marker Rhodopsin (Figures 4E,F, Supplementary Figures S10A,B″). The number of mature rods and L/M cones gradually increased with time increasing, up to D217 tested (Figures 4G,H, Supplementary Figures S10C,D″). In addition, functional proteins were also detected in the late-stage ROs. The α-subunit of rod transducin (Gα_t1_) was shown in eGFP+ cells from D120 (Figures 4I,J), while the cone-specific phototransduction protein CNGB3 was expressed in eGFP+ cells since D150 (Figures 4K,L). These results revealed that RCVRN-eGFP robustly labeled both mature cones and rods in late-stage ROs.

However, the eGFP+ cells did not express retinal progenitor markers VSX2, SIX3, and PAX6, or ganglion cell marker BRN3 in early-stage ROs (Supplementary Figure S11), indicating that RCVRN-eGFP did not label RPCs and ganglion cells. Moreover, the eGFP+ cells were also negative for amacrine cell marker AP2α, ganglion, amacrine, and horizontal cell marker HU C/D, müller glial cell marker CRALBP, and rod bipolar cell marker PKCα (Supplementary Figure S12), implying RCVRN-eGFP did not label these cells residing in the inner nuclear layer of the retina.

Collectively, the RCVRN-eGFP reporter covered nearly all developmental stages and all subtypes of photoreceptors, from photoreceptor precursors to mature cones and rods (Figure 4M), which could facilitate the selection and enrichment of photoreceptor cells at distinct stages.

### Transcriptome Pattern of the FACS-Sorted RCVRN-eGFP Positive Photoreceptors in ROs

**FIGURE 5:**
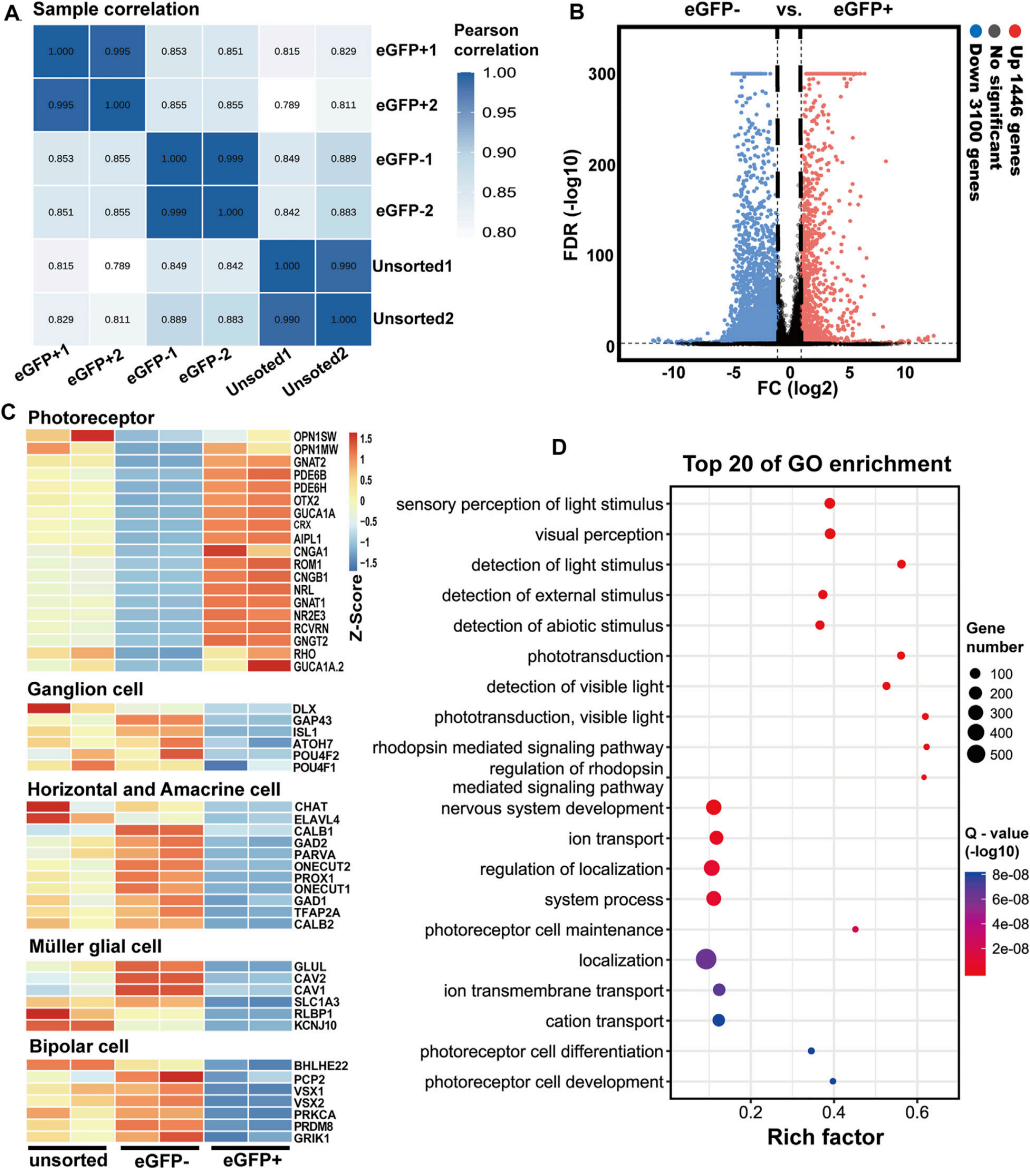
Transcriptome analysis of RCVRN-eGFP positive cells isolated from D150 retinal organoids by fluorescence-activated cell sorting. **(A)** Sample correlation of replicates of the unsorted, eGFP- and eGFP+ cells. **(B)** Volcano plots indicated fold change (FC) in gene expression between eGFP- and eGFP+ cells. Up, upregulated; Down, downregulated; vs., versus; FDR, false discovery rate. **(C)** Heat maps show gene expression patterns of known retinal cell-type-specific genes in the unsorted, eGFP- and eGFP+ cells. Blue to orange represented a gradient from low to high gene expression. **(D)** Bubble chart shows the top 20 enriched Gene Ontology (GO)-terms of biological processes in eGFP+ cells based on differentially expressed genes (DEGs) between eGFP- and eGFP+ cells. Rich factor (number of DEGs enriched in GO-terms/number of all genes in the background gene set). Color and size of the bubble represented enrichment significance and the number of DEGs enriched in GO-terms, respectively. Q-value < 0.05 was defined as significantly enriched.

We further investigated the transcriptome profile of RCVRN-eGFP positive cells in D150-ROs by RNA-seq. The eGFP positive and negative fractions were isolated and collected respectively using FACS (Supplementary Figure S13A). The percentage of eGFP+ cells in ROs was 43.36 ± 1.77% (N = 3 experiments), and the purity of the isolated cells in each fraction was more than 97.84%, which was evaluated by cell counting with a hemacytometer. Sample correlation analysis showed a high degree of correlation and reproducibility between the two independent sets (Figure 5A) in each group. Additionally, there were significant differences between the eGFP- and eGFP+ cells with alterations in 4,546 genes (Figure 5B).

Known retinal cell-type-specific genes were used to further identify the eGFP+ cells. The expression of photoreceptor-specific genes increased in eGFP+ cells such as *RCVRN, CRX, RHO*, and *OPN1MW*, consistent with the IF results (Figure 5C). In contrast, the expression of ganglion, horizontal, amacrine, müller glial, and bipolar cell marker genes decreased in eGFP+ cells (Figure 5C). Moreover, GO analysis showed the DEGs between eGFP- and eGFP+ cells were enriched in biological processes related to photoreceptor functions such as visual perception, detection of light stimulus, phototransduction, and detection of visible light (Figure 5D). Cellular components related to photoreceptor maturation and functions were also enriched such as photoreceptor cell cilium, photoreceptor outer segments, photoreceptor disc membrane, and synapse (Supplementary Figure S13B). All these data showed that the purified RCVRN-eGFP+ cells possessed transcriptome characteristics of photoreceptors, and the upregulation of photoreceptor marker genes in eGFP+ cells further demonstrated that photoreceptors could be well selected using the reporter line.

### Extraction of Potential CD Biomarkers for Photoreceptor Selection

**FIGURE 6:**
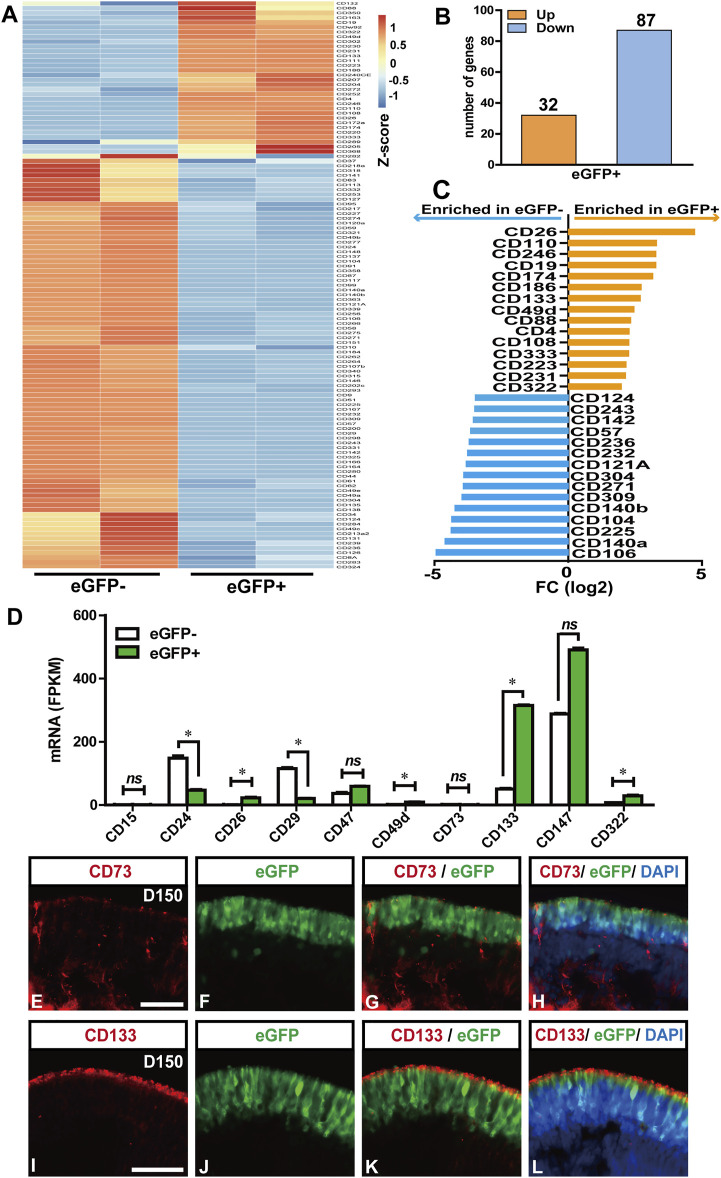
Potential clusters of differentiation (CD) biomarkers for RCVRN-eGFP+ photoreceptors. **(A)** Heat map showed all the differentially expressed genes (DEGs) of CD biomarkers between eGFP- and eGFP+ cells from D150 retinal organoids. Absolute fold change (FC) > 2 and false discovery rate (FDR) < 0.05 were considered significant. **(B)** Bar graph showed the number of upregulated (Up) and downregulated (Down) DEGs in eGFP+ cells, compared with eGFP- cells. **(C)** Bar graph showed the top 15 DEGs significantly upregulated with FPKM >1 in the eGFP+ photoreceptors (orange) and the top 15 DEGs upregulated with FPKM >1 in eGFP- cells (blue). **(D)** Bar graph showed the fragment per kilobase of transcript per million mapped reads (FPKM) values of the CD biomarkers reported previously for photoreceptor selection. *p < 0.05; ns, no significance (*p* > 0.05). **(E–H)** Immunofluorescence (IF) images showed CD73 was weakly and unspecifically expressed in the D150 reporter retinal organoids (ROs). **(I–L)** IF images verified the CD133 was specifically expressed in eGFP+ photoreceptors in the D150 ROs. Nuclei were stained with DAPI, Scale bars, 50 μm (E–L).

Although the FACS-sorted RCVRN-eGFP+ photoreceptors can be used as donor cells in animal models and promote preclinical research, they are not suitable as donor cells for clinical transplantation in treating patients. With the RNA-seq data of the eGFP positive and negative cells at D150, here, we attempted to analyze the mRNA expression of all the 394 CD biomarker genes listed in the gene group of CD molecules by the Human Genome Organization (HUGO) Gene Nomenclature Committee (HGNC) (https://www.genenames.org/data/genegroup/#!/group/471) in order to extract potential CD biomarkers for photoreceptor purification in the clinical settings. Differential expression analysis identified 121 DEGs of the CD biomarkers between the eGFP- and eGFP+ groups (Figure 6A). Compared with the eGFP- group, 32 DEGs were upregulated and 87 downregulated in the eGFP+ group (Figure 6B). The top 15 DEGs, which significantly upregulated with the FPKM >1 in the eGFP+ photoreceptors, were revealed, including CD26 (*DPP4*), CD110 (*MPL*), CD246 (*ALK*), CD19, and CD174 (*FUT3*) (Figure 6C). These genes could be potential CD biomarkers for the positive selection of photoreceptors. Meanwhile, the top 15 DEGs, which were significantly downregulated in the eGFP+ cells but upregulated with FPKM >1 in eGFP- cells, were also identified, including CD106 (*VCAM1*), CD140a (*PDGFRA*), CD225 (*IFITM1*), CD104 (*ITGB4*), and CD140b (*PDGFRB*), which could be potential CD biomarkers for negative selection of photoreceptor (Figure 6C).

In addition, CD biomarkers previously reported for the selection of mouse or human photoreceptors were further analyzed for their specificity and expressive abundance in the D150 FACS-sorted cells (Lakowski et al., 2015; Welby et al., 2017; Lakowski et al., 2018) (Figure 6D). Surprisingly, the mRNA expression of CD73 (*NT5E*), a widely used CD biomarker for positive selection of photoreceptor precursors and mature rods in mice and human retina (Koso et al., 2009; Eberle et al., 2011; Lakowski et al., 2015; Reichman et al., 2017; Gagliardi et al., 2018; Lidgerwood et al., 2018), actually had no significant difference between the eGFP- and eGFP+ cells (Figure 6D), which was further confirmed in the protein level by IF staining in D150 ROs (Figures 6E–H). These data were also consistent with previous studies on hiPSC/ESC-derived ROs (Welby et al., 2017; Lakowski et al., 2018). However, our results confirmed that the mRNA expression of CD133 (*PROM1*), a biomarker reported purifying photoreceptors in combination with other CD biomarkers (Lakowski et al., 2015; Welby et al., 2017), was highly and significantly expressed in the eGFP+ cells, compared to the eGFP- cells. The specificity of CD133 was further confirmed by IF analysis with D150 ROs (Figure 6I-L). These results indicated that the CD133 exclusively labeled eGFP+ photoreceptors should be suitable for photoreceptor selection.

## Discussion

Here, we established a photoreceptor-specific reporter hiPSC line by introducing the *eGFP* sequence into an endogenous *RCVRN* locus using CRISPR/Cas9 gene editing. The RCVRN-eGFP reporter faithfully recapitulated the endogenous expression of human recoverin during retinal differentiation. The RCVRN-eGFP initially appeared in the postmitotic photoreceptor precursors in early-stage ROs, and steadily presented in mature cones and rods at the late stage, nearly covering the whole developing stages and all subtypes of photoreceptors, which can help promote the positive selection of photoreceptors at distinct developmental stages. The transcriptome profiles of FACS-sorted RCVRN-eGFP+ cells at the late stage further revealed these purified cells possessed specific molecular signatures of photoreceptor cells. Importantly, potential CD biomarkers for photoreceptors were extracted from the RNA-seq data. Altogether, the reporter hiPSCs will provide a powerful tool for more extensive investigations and applications of human photoreceptors, especially cell enrichment and transplantation for outer retinal disorders.

Fluorescent proteins have been widely used for studying gene expression and visualizing cells in living animal models and hPSC reporter lines (Kaewkhaw et al., 2015; Collin et al., 2016; Smiley et al., 2016; Zelinger et al., 2017; Scott et al., 2019). They provide a powerful tool to understand real protein function, facilitate the selection and purification of specific cells. In transgenic mice with rod-specific expression of GFP, over 95% of donor photoreceptors could be efficiently enriched by flow cytometry. In addition, the survival and migration of these donor cells could be monitored by GFP after transplantation (MacLaren et al., 2006; Lakowski et al., 2011; Pearson et al., 2012; Gonzalez-Cordero et al., 2013). Similarly, several photoreceptor-specific reporter hiPSC/ESC lines have been established. For example, two CRX reporter lines were constructed and applied for the development monitor, transcriptome profiling, cell isolation, and transplantation of the photoreceptor precursors (Kaewkhaw et al., 2015; Collin et al., 2016; Collin et al., 2019). The NRL reporter line and L/M opsin reporter line were also generated and used for tracking the human rod differentiation (Phillips et al., 2018a) and isolation of cones (Welby et al., 2017), respectively.

However, in the late stage of RDs, all subtypes of photoreceptors are lost, and both rods and cones are needed for graft. Especially, accumulating evidence has indicated that ontogenetic stages of donor photoreceptors critically influence the success of transplantation, including cell survival, migration, integration, and even vision recovery (Santos-Ferreira et al., 2017; Llonch et al., 2018). Although some studies have suggested that postmitotic photoreceptor precursors would be preferred (MacLaren et al., 2006; Pearson et al., 2012; Singh et al., 2013; Santos-Ferreira et al., 2015), there is no agreement about which developmental stage of photoreceptor cells is optimal for transplantation with the best outcome in the field. It is necessary to acquire abundant donor cells, at different developmental stages, in particular, to evaluate their impact on cell therapy. In this study, we generated an RCVRN-eGFP reporter hiPSC line, which could unbiasedly label both cones and rods. Importantly, another advantage of the reporter was that the RCVRN-eGFP specifically and robustly covered nearly all the developmental stages of photoreceptors from the photoreceptor precursors to mature cones and rods. Therefore, in combination of the expression pattern of RCVRN-eGFP reporter and a given time point of retinal differentiation, photoreceptors at distinct ontogenic stages could be selected and purified from the reporter ROs, including photoreceptor precursors before D90, immature photoreceptors between D90-D150 and relatively mature photoreceptors after D150. Moreover, RNA-seq analysis showed that the eGFP+ cells sorted by FACS had typical transcriptome characteristics of photoreceptors, further confirming the high fidelity and the favorable purification effect of the RCVRN-eGFP reporter in photoreceptor cells. Collectively, the successful generation of the RCVRN-eGFP reporter hiPSCs can provide a great number of photoreceptor cells as donors, at distinct ontogenic stages in particular, for the evaluation of cell transplantation in RD animal models, and thus promote the development of novel cell therapy for patients with advanced RDs.

Recoverin is an important Ca^2+^-binding protein and plays a key role in phototransduction (Morshedian et al., 2018). In recoverin-deficient mice, the lifetime of light-activated rhodopsin was reduced and the decay of rod light response was accelerated (Makino et al., 2004; Sampath et al., 2005; Chen et al., 2010; Chen et al., 2012). The sensitivity of rod-mediated vision was reduced in recoverin-deficient rods (Sampath et al., 2005). Likewise, the photosensitivity of recoverin-deficient cones decreased (Sakurai et al., 2015). Our study observed that the rod- and cone-specific phototransduction proteins, Gα_t1_ for rods and CNGB3 for cones, could be detected and co-labeled with eGFP in the late stage of the RCVRN-reporter ROs. The outer segment-like protrusions, a functional structure, appeared on the surface of ROs and remained apparent up to D217, the longest time point observed in this study. Furthermore, GO analysis disclosed that the biological processes, related to photoreceptor functions such as visual perception, detection of light stimulus, phototransduction, and detection of visible light, were enriched in the eGFP+ cells isolated from D150 ROs by FACS. Cellular components related to photoreceptor maturation and functions were also enriched in the eGFP+ cells, including photoreceptor cell cilium, photoreceptor outer segments, photoreceptor disc membrane, and synapse. All these findings revealed that the RCVRN-reporter ROs could obtain advanced maturation and express functional proteins or mRNAs, implying the potential in disease modeling and drug screening of RDs such as RP and AMD.

Recently, stem cell-based therapies have become a promising treatment for retinal disorders with photoreceptor defects, which aim to replace the dysfunctional photoreceptors in the host retina. One major hindrance preventing the clinical application of hPSCs-derived photoreceptors is the lack of strategies to purify safe and effective donor cells. Although the photoreceptor marker reporter lines including the RCVRN-reporter can serve as good tools to purify the specific photoreceptor cells for basic and pre-clinical investigations, these genetically tagged donor cells are not suitable for clinical therapy. Instead, cell surface markers have been successfully used to purify interested cells for clinical application with FACS or MACS, such as blood cells (Michael et al., 2013; Lamb et al., 2021). Several attempts have been made to explore cell surface markers specific for photoreceptors in mice and human retina for future clinical use (Postel et al., 2013; Kaewkhaw et al., 2015). For instance, Kaewkhaw et al. proposed several potential surface markers for photoreceptor selection by transcriptome analyses of CRX reporter human ROs younger than D90, recommending KCNV2 for photoreceptors, RTN4RL1, ST3GAL5, GNGT2, and EPHA10 for cones, GABRR2 and CNGB1 for rods (Kaewkhaw et al., 2015). However, the specificity of these surface markers was not further confirmed.

CD biomarkers have become the most widely used cell surface markers for basic and clinical applications since their antibodies are commercially available and well defined (Uder et al., 2018; Haybar et al., 2020). Panels of CD markers for tagging photoreceptors in mouse and human retina have been reported (Reichman et al., 2017; Welby et al., 2017; Lakowski et al., 2018). For example, CD73 has been used alone (Koso et al., 2009; Eberle et al., 2011; Gagliardi et al., 2018) or in combination (Lakowski et al., 2011; Lakowski et al., 2015) for the enrichment of photoreceptor precursors or mature rods. However, our RNA-seq data showed that its expression had no difference in RCVRN-eGFP+ and RCVRN-eGFP- cells, which was consistent with previous reports (Welby et al., 2017; Lakowski et al., 2018). Meanwhile, our results also revealed that CD133, which was reported for human cone enrichment when combined with CD147+/CD26+/CD15- (Welby et al., 2017), had significantly higher expression in the RCVRN-eGFP+ photoreceptors. Its specificity to exclusively label photoreceptors was also confirmed by IF analysis. Therefore, CD133 would serve as a favorable biomarker for the enrichment of human photoreceptors. Furthermore, in all the 394 CD biomarkers analyzed between the eGFP- and eGFP+ cells isolated from the D150 reporter ROs, 15 potential DEGs for positive selection of photoreceptors were recommended, such as CD26, CD110, CD246, CD19, CD147, and CD133, while 15 potential DEGs for negative selection of photoreceptors were also suggested, such as CD106, CD140a, CD225, CD104, and CD140b. Although these CD biomarker genes required further validation experiments, they provided an important data foundation for future optimization, and thus would promote the cell replacement therapies of RDs.

In conclusion, we successfully generated an RCVRN-eGFP reporter hiPSC line and firstly revealed the molecular signatures of recoverin+ photoreceptors in ROs derived from this reporter. The RCVRN-eGFP faithfully monitored the endogenous expression of human recoverin during retinal development in ROs. The reporter ROs can be used as live-tracking models for retinal photoreceptor development, and a stable and efficient tool for the enrichment of photoreceptors at all the developmental stages to facilitate the evaluation of cell therapy for RDs. Potential CD biomarkers for positive or negative selection of photoreceptors were also extracted in this study, which will benefit clinical applications in cell regenerative studies.

## Data Availability

The datasets presented in this study can be found in online repositories. The names of the repository/repositories and accession number(s) can be found below: https://www.ncbi.nlm.nih.gov/geo/, GSE196355.
