# Supplementary material for: Generation of an RCVRN-eGFP Reporter hiPSC Line by CRISPR/Cas9 to Monitor Photoreceptor Cell Development and Facilitate the Cell Enrichment for Transplantation
Source: Front Cell Dev Biol. 2022 Apr 28;10:870441. doi: 10.3389/fcell.2022.870441 (PMC9096726; doi:10.3389/fcell.2022.870441)
Supplement: Supplementary file 1 [file DataSheet1.docx]

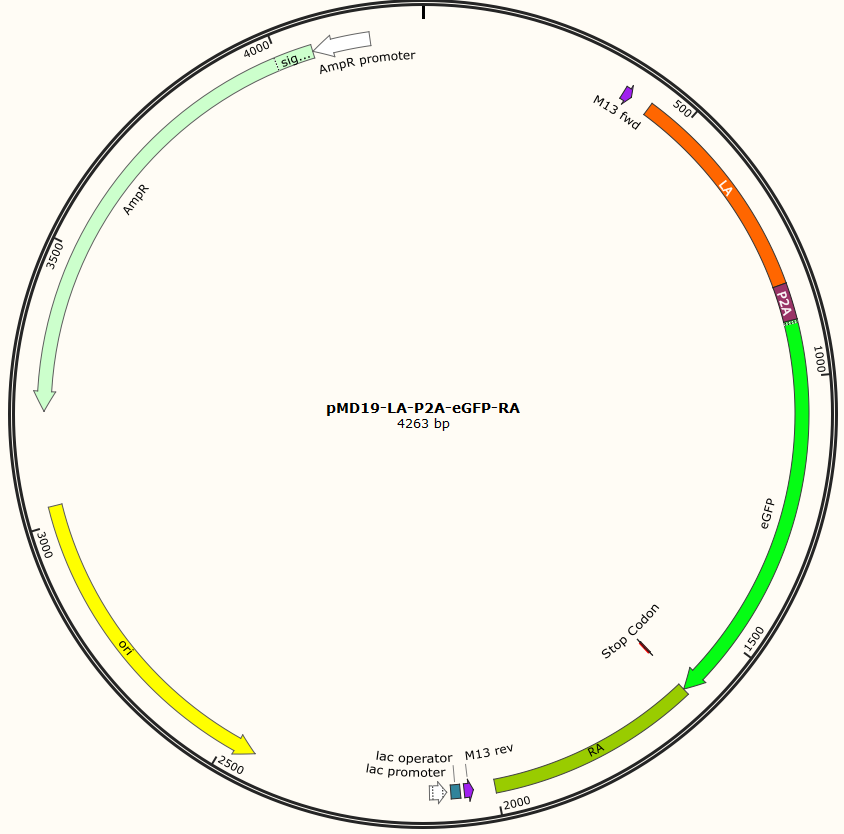
*Supplementary Material*

**Supplementary Figure S1. Donor plasmid map.** *P2A* self-cleaving peptide sequence from porcine teschovirus was fused with *eGFP* coding sequence, followed by the TAA stop codon at the end of *eGFP*. *RCVRN* homologous arms LA and RA flanked the either side of *P2A-eGFP*. LA, left homologous arm; P2A, porcine teschovirus 2A self-cleaving peptide; eGFP, enhanced green fluorescence protein; RA, right homologous arm.

**
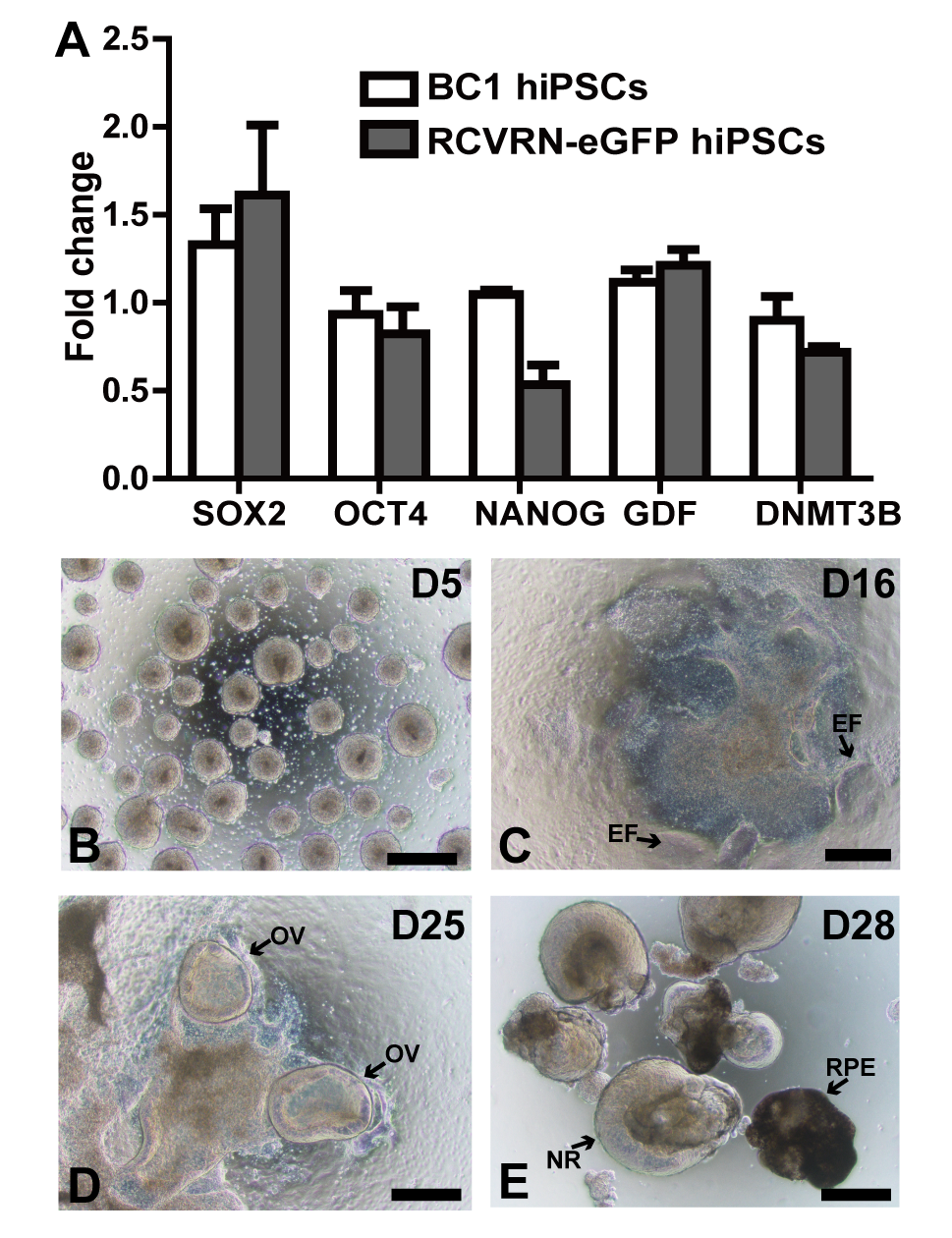
**

**Supplementary Figure S2. Characterization of the** **pluripotency and retinal differentiation ability of the RCVRN-eGFP reporter hiPSCs**. **(A)** Quantitative reverse transcription-polymerase chain reaction (qRT-PCR) showed that there was no significant difference between the parental and reporter hiPSCs in the expression of the pluripotency markers SOX2, OCT4, NANOG, GDF and DNMT3B. hiPSCs, human induced pluripotent stem cells; eGFP, enhanced green fluorescent protein **(B)** Bright field of embryoid bodies. **(C)** Bright field of Eye Field (EF) domains. **(D)** Bright field of optical vesicles (OVs). **(E)** Bright field of retinal organoids (ROs) with NR layers and retinal pigment epithelium cells (RPEs). Scale bars, 100 μm **(B-E)**.


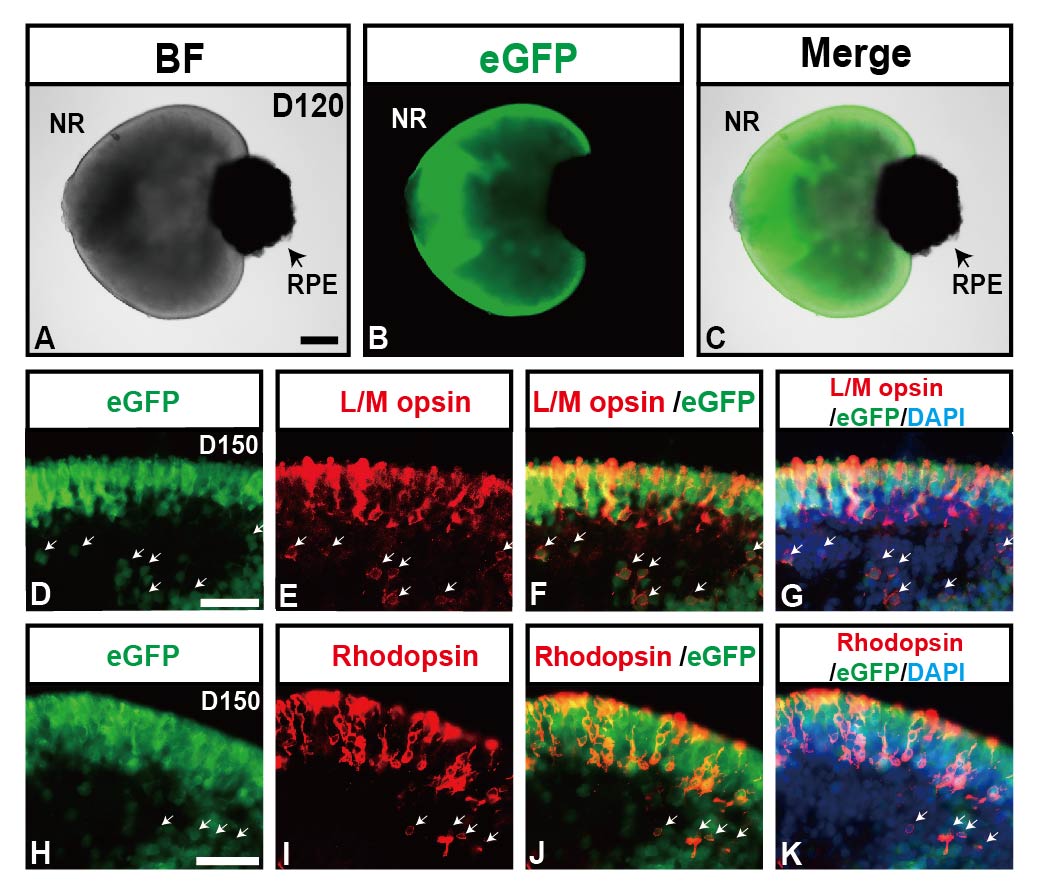


**Supplementary Figure S3. The expression of RCVRN-eGFP in the reporter retinal organoids. (A-C)** Bright field (BF) and fluorescent images showed that RCVRN-eGFP was expressed in NR layer of ROs, but not in RPE. eGFP: enhanced green fluorescence protein; NR: neural retina; RPE: retinal pigment epithelium. Scale bars, 200 μm **(A-C)**.


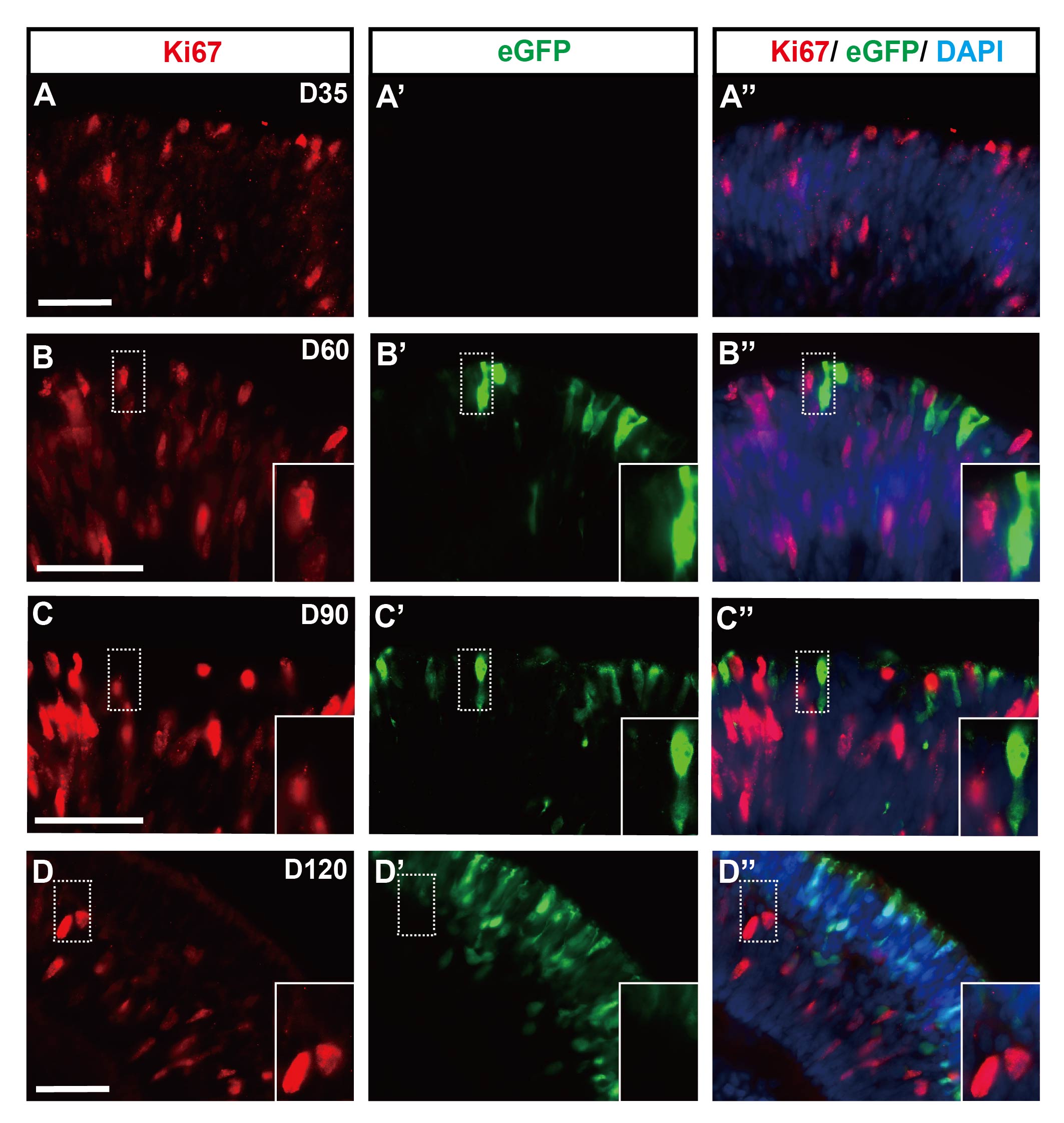


**Supplementary Figure S4. (A-D’’)** Immunofluorescence images related to **Figure 3A-D** showed RCVRN-eGFP+ cells did not express the proliferation marker Ki67 in the reporter retinal organoids (ROs) at different timepoints. Nuclei were stained with DAPI. Scale bars, 50μm **(A-D’’)**.


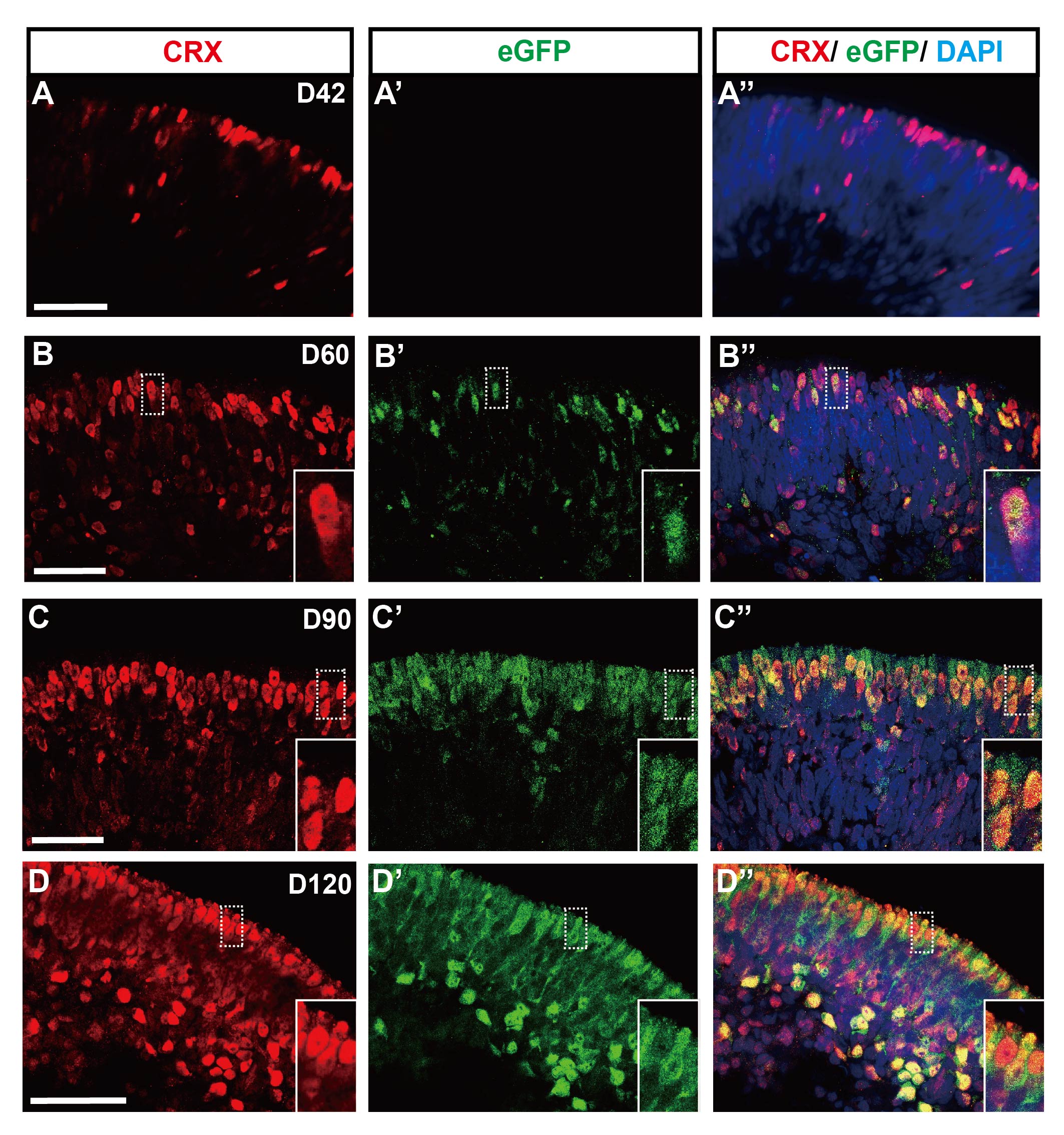


**Supplementary Figure S5.** **(A-D’’)** Immunofluorescence images related to **Figure 3E-H** showed the time-course expression of RCVRN-eGFP and photoreceptor precursor marker CRX in early stage ROs younger than D120. Nuclei were stained with DAPI. Scale bars, 50μm **(A-D’’)**.


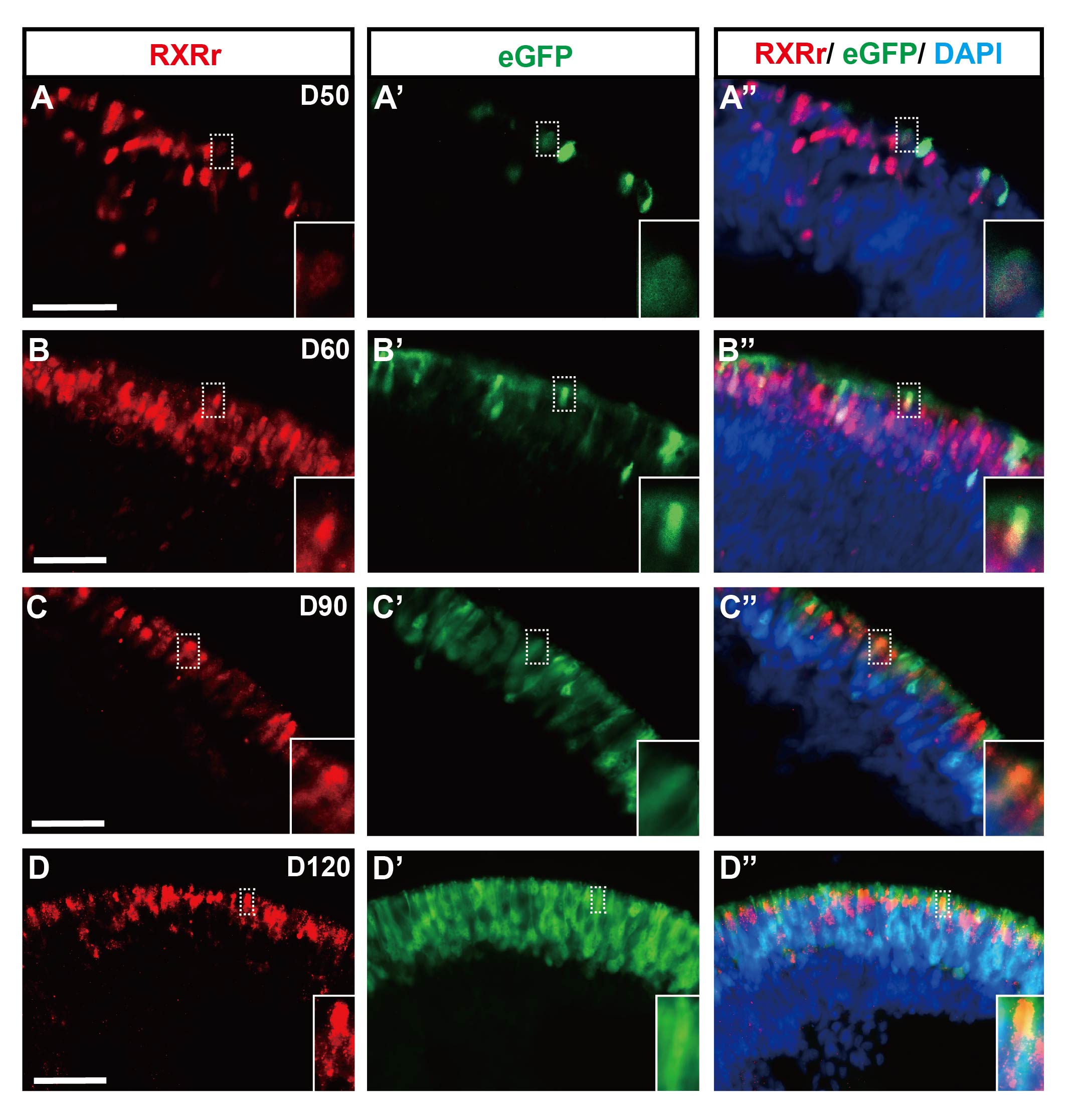


**Supplementary Figure S6.** **(A-D’’)** Immunofluorescence images related to **Figure 3I-L** showed the time-course expression of cone precursor marker RXRr and the reporter eGFP in early stage reporter ROs younger than D120. Nuclei were stained with DAPI. Scale bars, 50μm **(A-D’’)**.


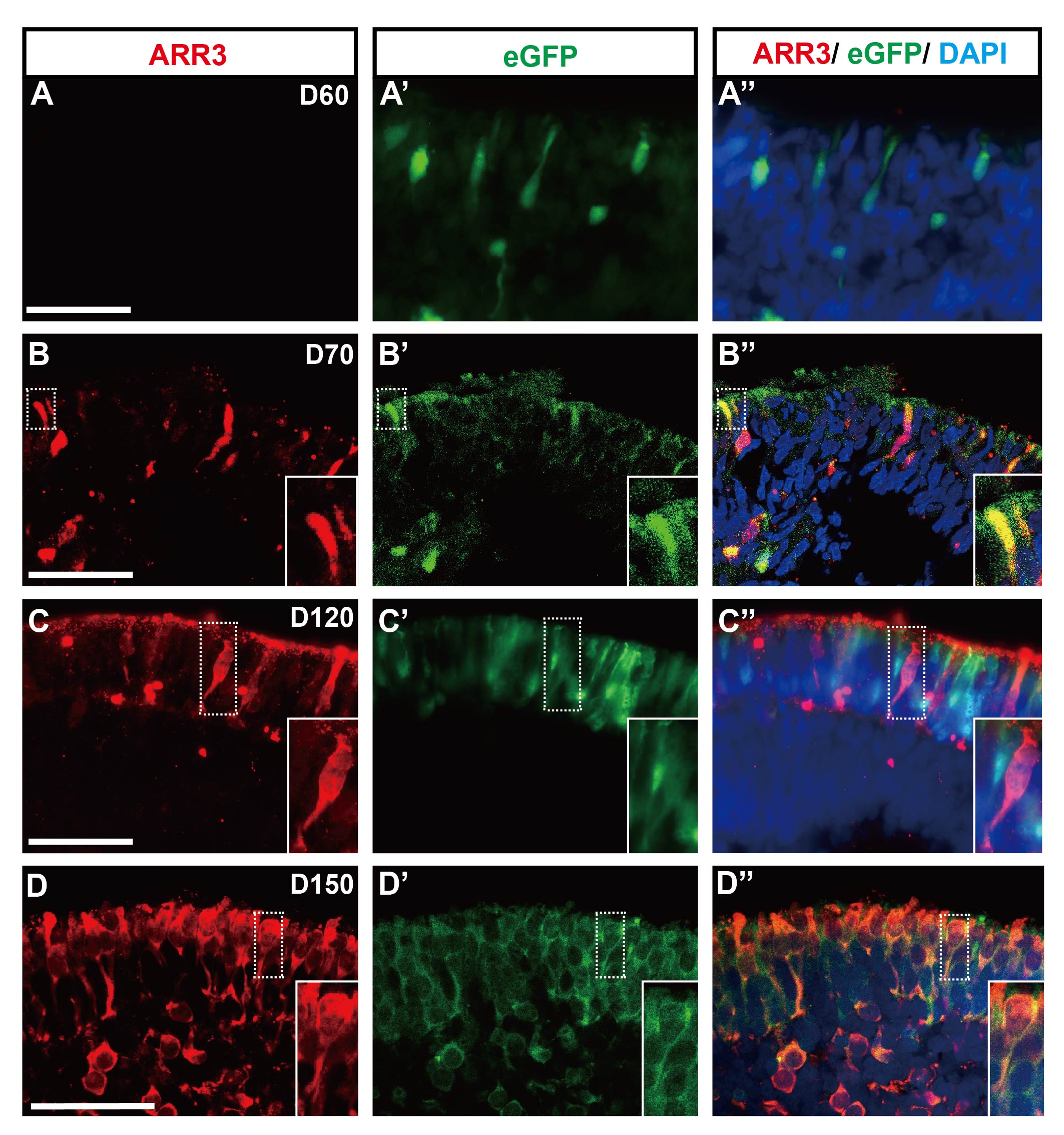


**Supplementary Figure S7.** **(A-D’’)** Immunofluorescence images related to **Figure 3M-P** showed the time-course expression pattern of cone precursor marker ARR3 and the reporter eGFP in early stage reporter ROs younger than D120. Nuclei were stained with DAPI. Scale bars, 50μm **(A-D’’)**.


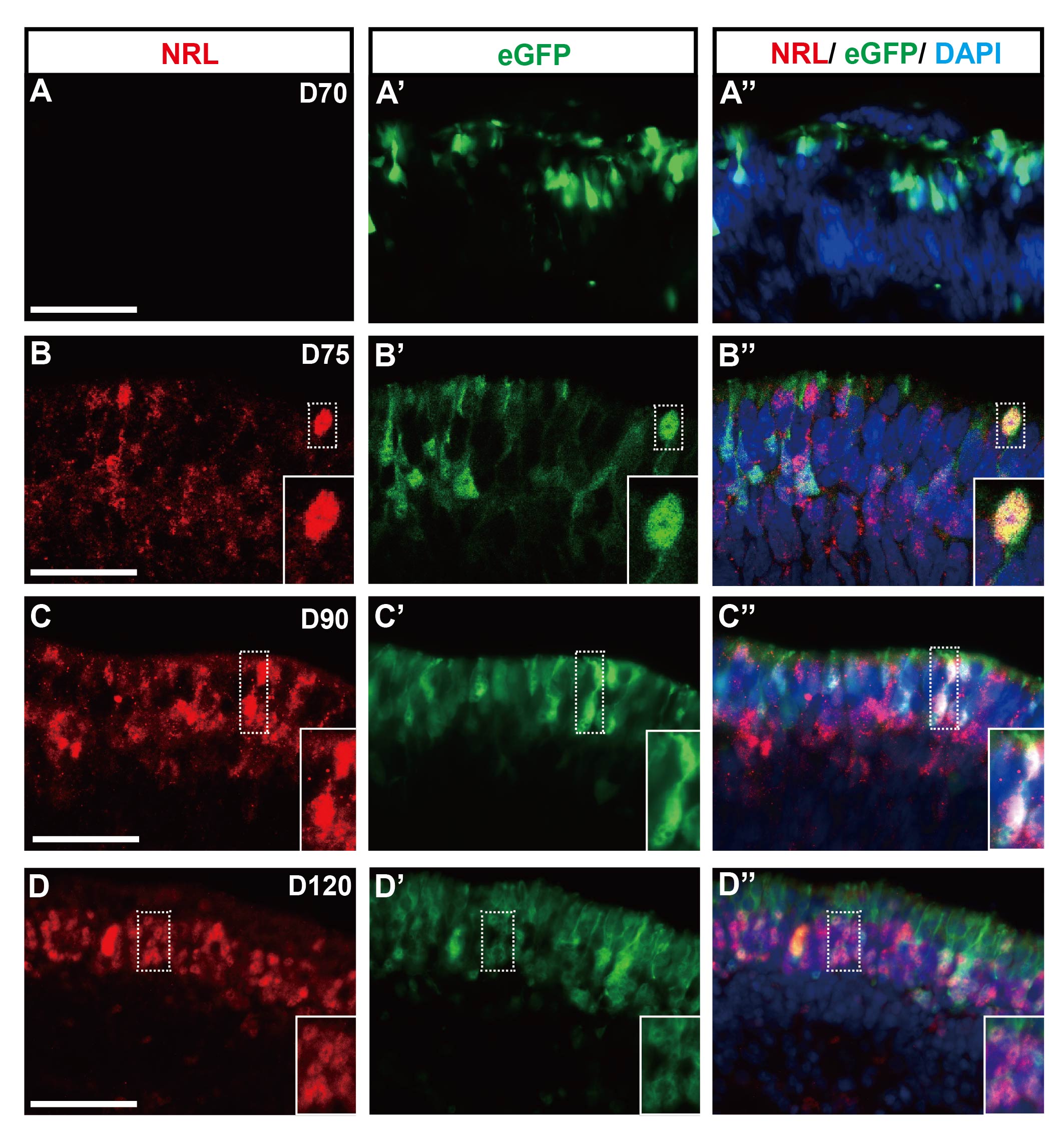


**Supplementary Figure S8.** **(A-D’’)** Immunofluorescence images related to **Figure 3Q-T** showed the time-course expression pattern of rod precursor marker NRL and the reporter eGFP in early stage ROs younger than D120. Nuclei were stained with DAPI. Scale bars, 50μm **(A-D’’)**.


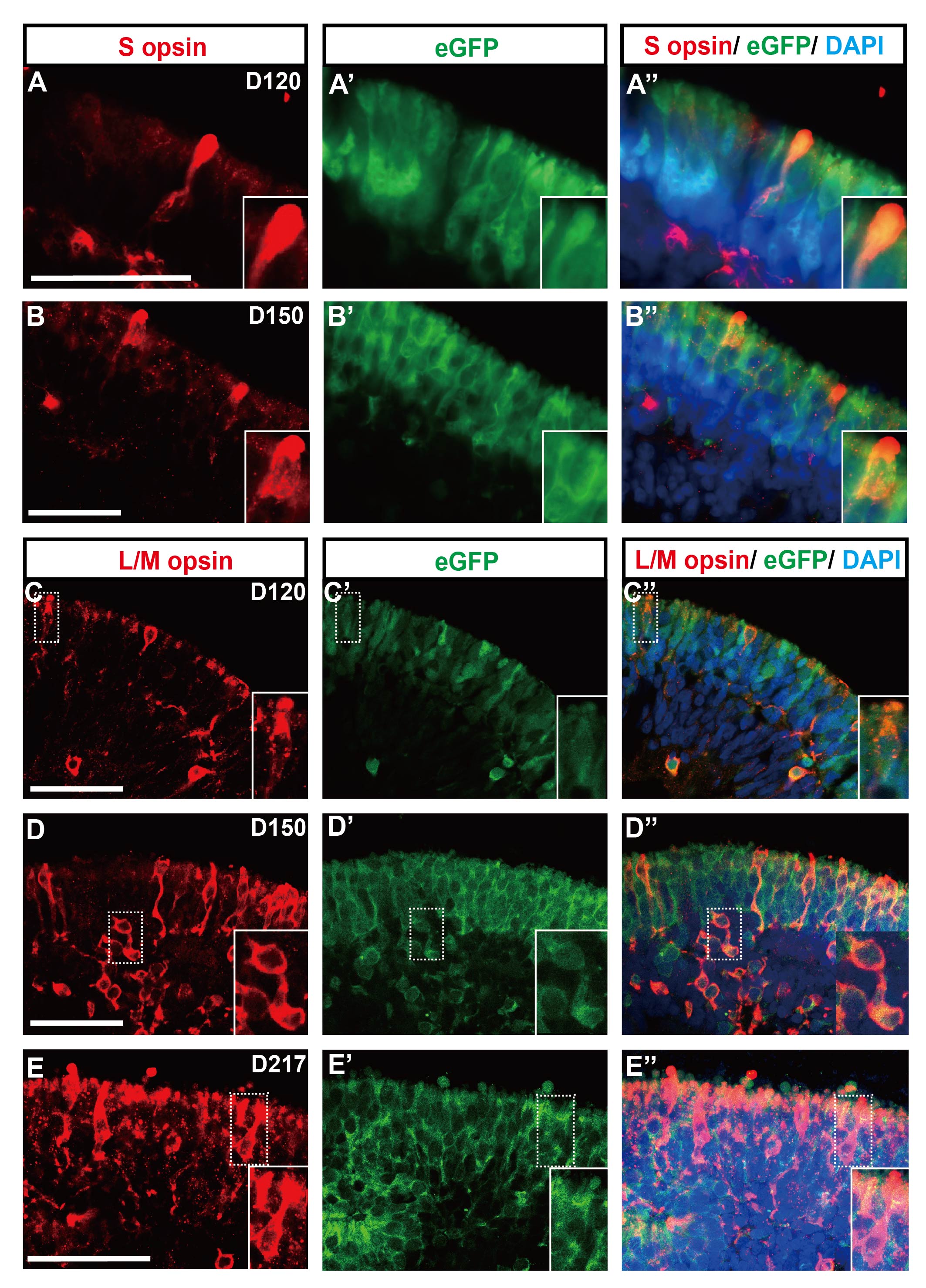


**Supplementary Figure S9.** (**A-D’’)** Immunofluorescence images related to **Figure 4A-D** showed a subpopulation of eGFP+ cells expressed blue cone marker S opsin and red/green cone marker L/M opsin in late stage retinal organoids older than D120. Nuclei were stained with DAPI. Scale bars, 50μm **(A-D’’)**.


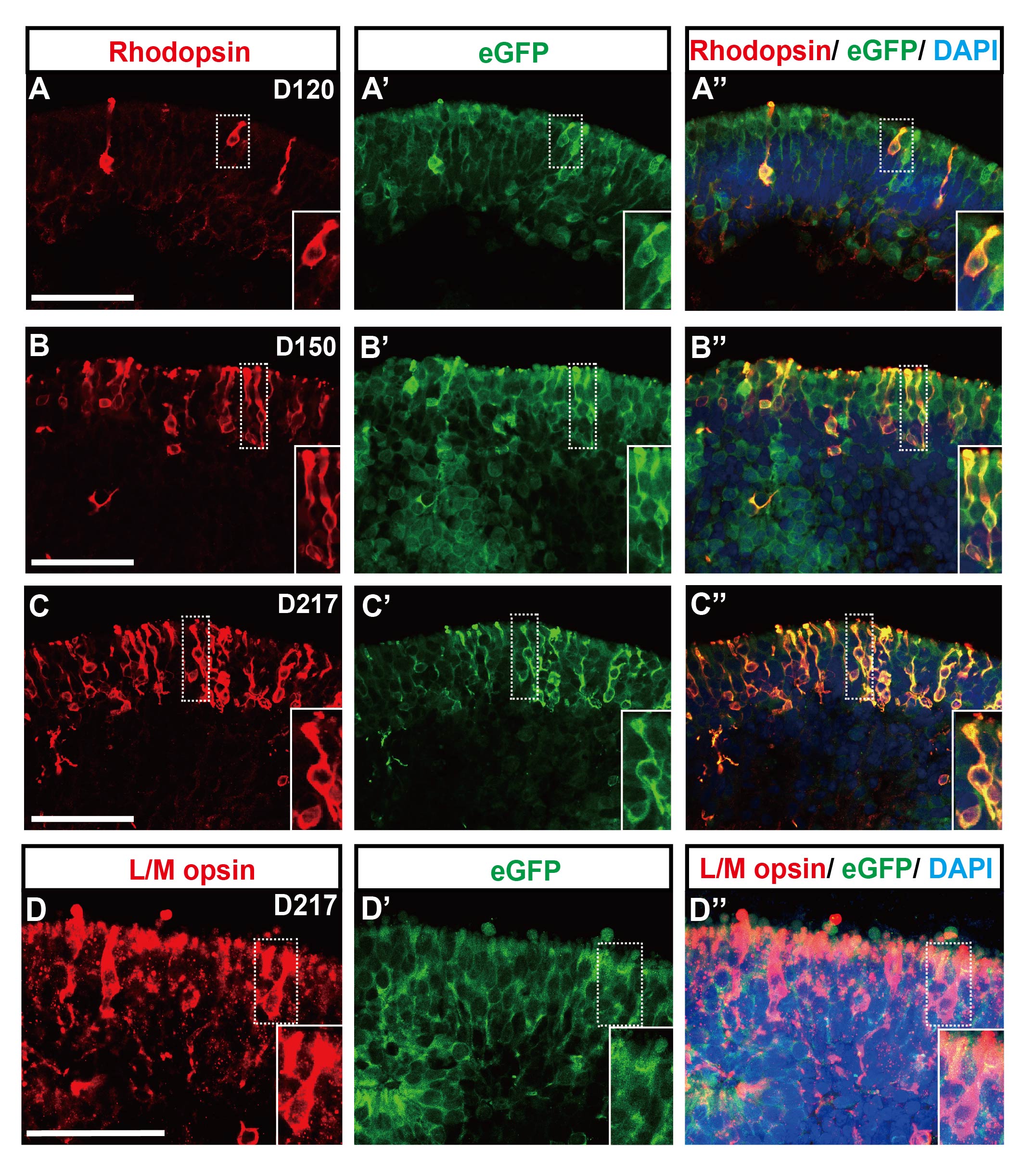


**Supplementary Figure S10. (A-B’’)** Immunofluorescence (IF) images related to **Figure 4E-H** showed the expression of rod marker Rhodopsin in late stage retinal organoids older than D120. **(C-D’’)** IF images showed the number of mature rods and L/M cones gradually increased with time increasing, up to D217 tested. Nuclei were stained with DAPI. Scale bars, 50μm **(A-D’’)**.


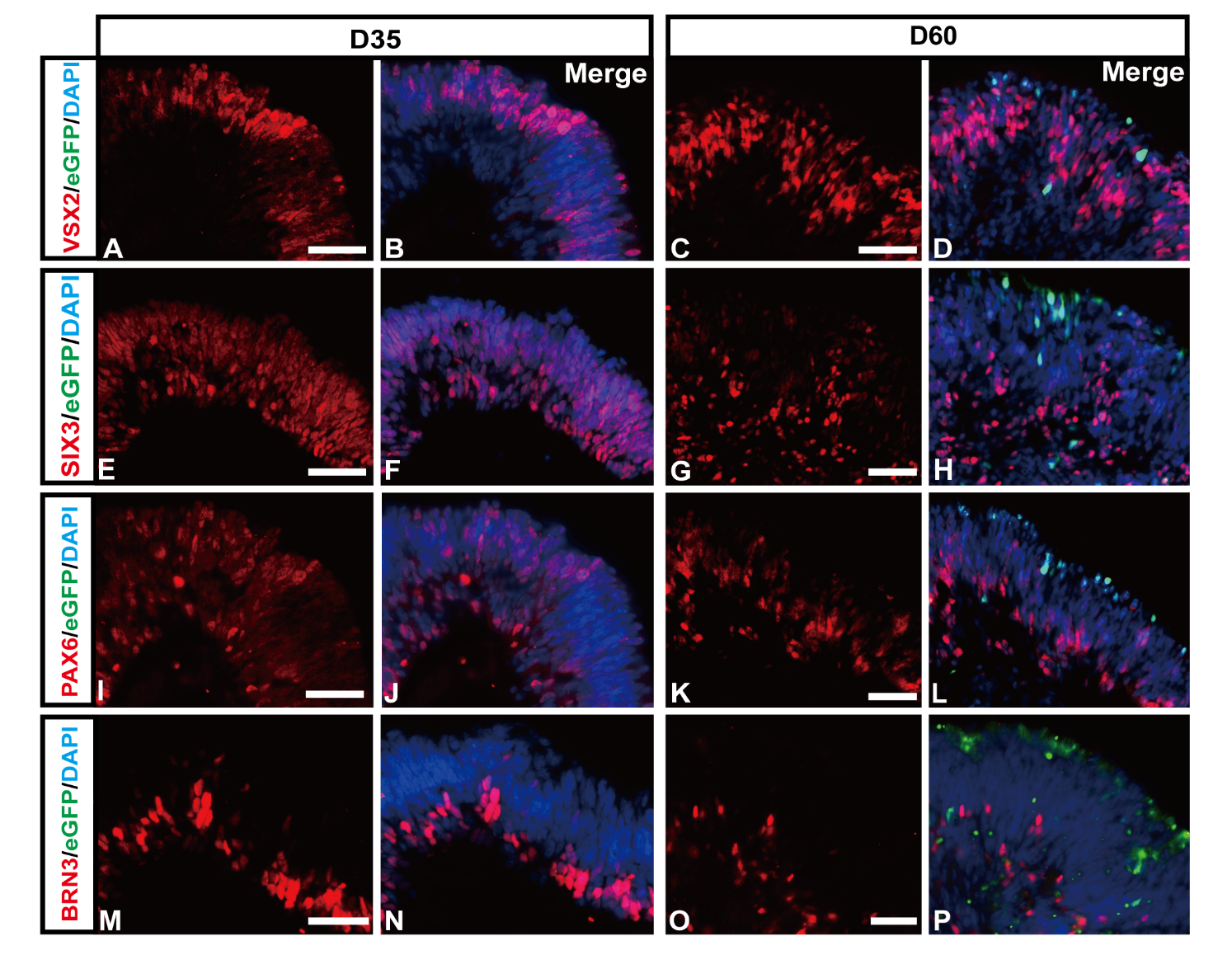


**Supplementary Figure S11. RCVRN-eGFP+ cells did not express retinal progenitor cell markers and ganglion cell marker. (A-P)** Immunofluorescence images showed RCVRN-eGFP had no co-expression with retinal progenitor cell markers VSX2, SIX3 and PAX6, and ganglion cell marker BRN3 in retinal organoids aged at D35 and D60 after differentiation. Nuclei were stained with DAPI. Scale bars, 50 μm **(A-P)**.


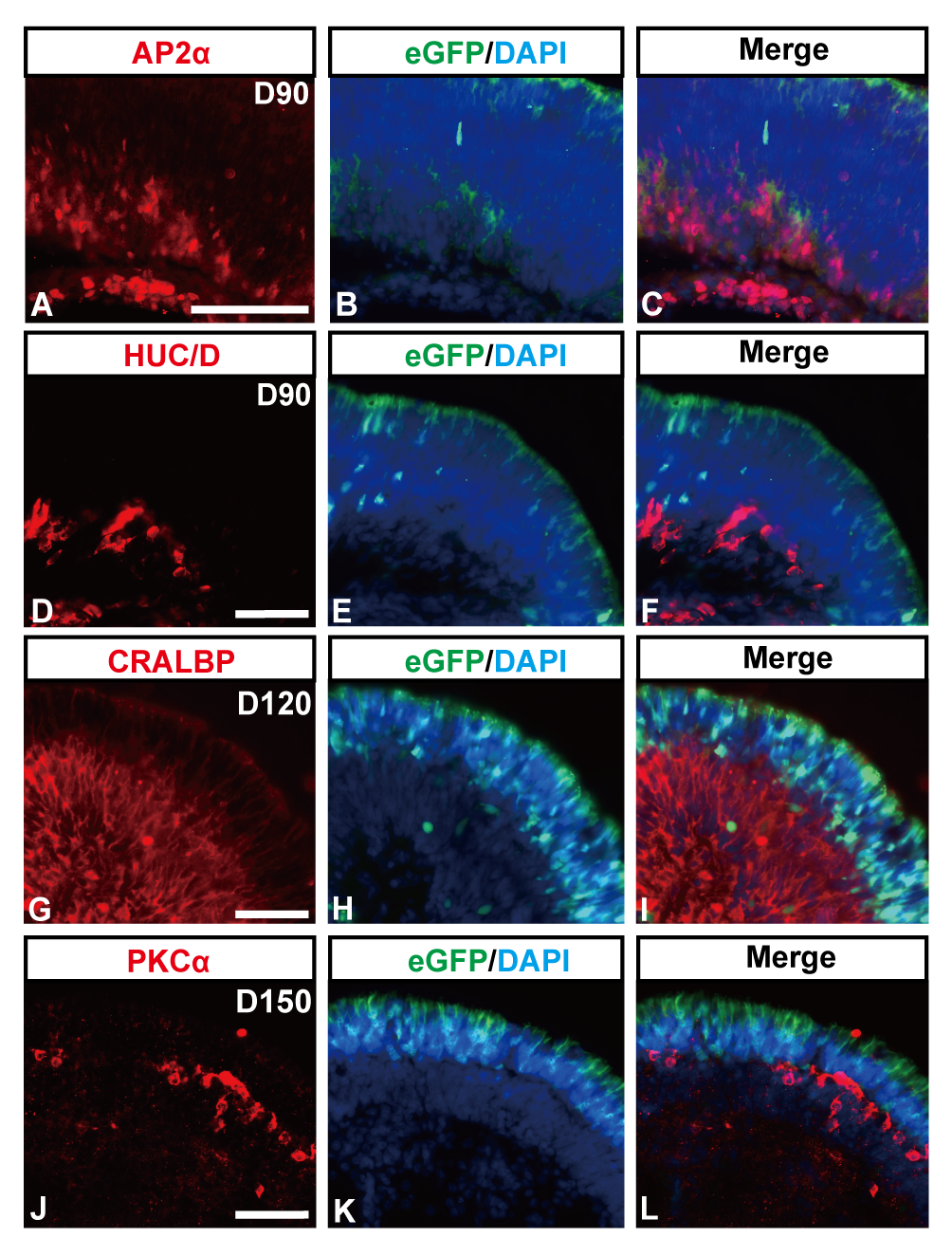


**Supplementary Figure S12. RCVRN-eGFP+ cells did not express amacrine cell, horizontal cell, Müller glial cell and bipolar cell markers. (A-L)** Immunofluorescence images showed eGFP+ cells had no expression of amacrine cell marker AP2α, ganglion, amacrine and horizontal cell marker HU C/D, Müller glial cell marker CRALBP and bipolar cell marker PKCα in the reporter retinal organoids. Nuclei were stained with DAPI. Scale bars, 50 μm **(A-L)**.


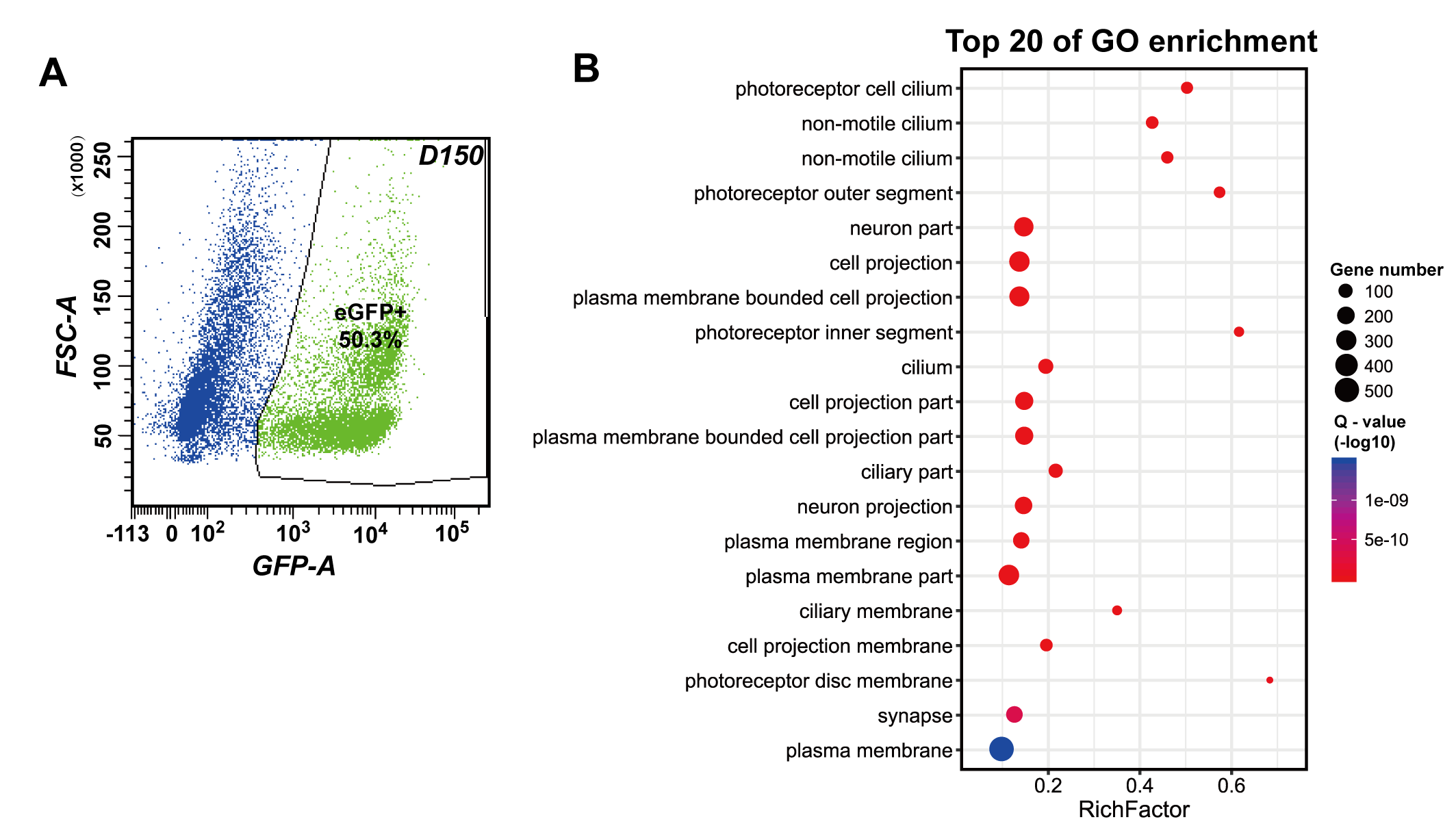


**Supplementary Figure S13. Enrichment and transcriptome profile of RCVNR-eGFP positive cells. (A)** Dot plot showed eGFP+ cells were sorted from the D150 reporter retinal organoids by fluorescence-activated cell sorting. Cell debris and dead cells were excluded. FSC-A, forward scatter-area; eGFP, enhanced green fluorescent protein. **(B)** Bubble chart showed top 20 enriched Gene Ontology (GO)-terms of cellular components in eGFP+ cells based on differentially expressed genes (DEGs) between eGFP- and eGFP+ cells by GO enrichment analysis. RichFactor (number of DEGs enriched in GO-terms / number of all genes in the background gene set). Color represented enrichment significance, and the size of the bubble represented the number of DEGs enriched in GO-terms. Q-value < 0.05 was defined as significant enriched.

**Supplementary Table 1: PCR primers for targeting plasmid construction**

| **Primer name** | **Primer sequence (5’ → 3’)** | **Primer length**  **(bp)** |
| --- | --- | --- |
| LA | F: GGCCAATCGGAAGAGACACATAGC | 24 |
|  | R: GGCGTTCTTCATCTTTTCCTTCACT | 25 |
| RA | F: TGCCAACTGTTCAGCTGTCCT | 21 |
|  | R: CCCAGCTCAGCTTGCGTTTATTG | 23 |
| P2A-eGFP | F: GGAAGCGGAGCTACTAACTTCAGC | 24 |
|  | R: TTACTTGTACAGCTCGTCCATGCC | 24 |
| IFSN_LA | F: GAGCTCGGTACCCGGGGATCGGCCAATCGGAAGAGACACA | 40 |
|  | R: CTCCGCTTCCGGCGTTCTTCATCTTTTCCTTCAC | 34 |
| IFSN_RA | F: GTACAAGTAATGCCAACTGTTCAGCTGTCC | 30 |
|  | R: CAGGTCGACTCTAGAGGATCCCCAGCTCAGCTTGCGTTTATTG | 43 |
| IFSN_P2A-eGFP | F: GAAGAACGCCGGAAGCGGAGCTACTAACTTCAGC | 34 |
|  | R: ACAGTTGGCATTACTTGTACAGCTCGTCCATGCC | 34 |

**Note:** F, forward primer; R, reverse primer; LA, left homologous arm; RA, right homologous arm; IFSN, in-fusion. The underlined parts indicate in-fusion site.

**Supplementary Table2: List of antibodies used for immunofluorescence staining**

| **Antibody name** | **Company** | **Catalog number** | **Host** | **Dilution** |
| --- | --- | --- | --- | --- |
| **First antibody** | | | | |
| AP2α | DSHB | 3B5-S | Mouse | 1/35 |
| ARRESTIN3(ARR3) | Novus | NBP1-37003 | Goat | 1/200 |
| BRN3 | Santa cruz | sc-6026 | Goat | 1/100 |
| CD133 | Abcam | ab19898 | Rabbit | 1/1000 |
| CD73 | Biolegend | 344002 | Mouse | 1/100 |
| CNGB3 | absin | abs121693 | Rabbit | 1/800 |
| CRALBP | Abcam | ab15051 | Mouse | 1/500 |
| CRX | Abnova | H00001406-M02 | Mouse | 1/800 |
| Gα _t1_ | Santa cruz | sc-136143 | Mouse | 1/2000 |
| HU C/D | Santa cruz | sc-48421 | Mouse | 1/200 |
| Ki67 | Abclonal | A11390 | Rabbit | 1/200 |
| L/M opsin | Gift from Dr Jeremy Nathans | | Rabbit | 1/5000 |
| NANOG | Abcam | ab21624 | Rabbit | 1/1000 |
| NRL | Santa cruz | sc-374277 | Mouse | 1/200 |
| OCT4 | Abclonal | A7920 | Rabbit | 1/200 |
| PAX6 | DSHB | 3B5 | Mouse | 1/50 |
| PKCα | Abcam | ab32376 | Rabbit | 1/2000 |
| Recoverin | Millipore | ab5585 | Rabbit | 1/500 |
| Rhodopsin | Abcam | ab5417 | Mouse | 1/200 |
| RXRr | Abcam | ab15518 | Rabbit | 1/200 |
| S opsin | Gift from Dr Jeremy Nathans | | Rabbit | 1/5000 |
| SIX3 | Rockland | 600-401-A26 | Rabbit | 1/500 |
| SSEA4 | Abcam | ab16287 | Mouse | 1/100 |
| TRA-1-60 | Abcam | ab16288 | Mouse | 1/400 |
| VSX2 | Millipore | ab9016 | Sheep | 1/200 |
| **Second antibody** | | | | |
| Donkey Anti-Mouse IgG-555 | Invitrogen | A31570 | Mouse | 1/500 |
| Donkey Anti-Rabbit IgG-555 | Invitrogen | A31572 | Rabbit | 1/500 |
| Donkey Anti-Goat IgG-555 | Invitrogen | A21432 | Goat | 1/500 |
| Donkey Anti-Sheep IgG-555 | Invitrogen | A21436 | Sheep | 1/500 |
| Donkey Anti-Rabbit IgG-647 | Invitrogen | A31573 | Rabbit | 1/500 |
| Donkey Anti-Mouse IgG-647 | Invitrogen | A31571 | Mouse | 1/500 |
| **Nuclei** | | | | |
| DAPI | Dojindo | D523 | - | 1/1000 |

**Supplementary Table 3: List of primers for qRT-PCR^a^**

| **Genes** | **Size (bp)** | **Forward** | **Reverse** |
| --- | --- | --- | --- |
| *DNMT3B* | 203 | ATAAGTCGAAGGTGCGTCGT | GGCAACATCTGAAGCCATTT |
| *GAPDH* | 87 | TGCACCACCAACTGCTTAGC | GGCATGGACTGTGGTCATGAG |
| *GDF3* | 179 | AAATGTTTGTGTTGCGGTCA | TCTGGCACAGGTGTCTTCAG |
| *NANOG* | 237 | AAGGTCCCGGTCAAGAAACAG | CTTCTGCGTCACACCATTGC |
| *OCT4* | 127 | AACCCACACTGCAGCAGATCA | TCTCGTTGTGCATAGTCGCT |
| *SOX2* | 176 | GACAGTTACGCGCACATGAA | TAGGTCTGCGAGCTGGTCAT |

^a^qRT-PCR: Quantitative Reverse Transcription-PCR
